# Neutron Transport and Layer-Resolved Radiation Effects in Potassium-Ion Cells with Organic and Inorganic Cathodes Under Space-Relevant Irradiation

**DOI:** 10.3390/nano16181188

**Published:** 2026-09-20

**Authors:** Ivan E. Novoselov, Ivan S. Zhidkov

**Affiliations:** 1 Institute of Physics and Technology, Ural Federal University, Mira 19 Street, Yekaterinburg 620062, Russia; 2 Federal Research Center of Problems of Chemical Physics and Medicinal Chemistry of the Russian Academy of Sciences, Semenov Av., 1, Chernogolovka 142432, Russia; 3 M.N. Mikheev Institute of Metal Physics, ral Branch of the Russian Academy of Sciences, S. Kovalevskoi 18 Street, Yekaterinburg 620108, Russia

**Keywords:** potassium-ion battery, TPHATP, LiFePO_4_, neutron transport, Geant4, Monte Carlo simulation, radiation response, space applications

## Abstract

The neutron response of potassium-ion batteries containing organic electrode materials remains insufficiently understood, particularly for applications in radiation environments. In this work, a multilayer Geant4 model of a CR2032 potassium-ion cell with a TPHATP composite cathode was developed and compared with a geometrically equivalent LiFePO_4_ reference model. Prompt fission neutron spectra from ENDF/B-VIII.0, JEFF-3.3, and JENDL-5 were used to evaluate neutron transmission, reflection, layer-resolved energy deposition, NIEL, secondary particle production, and displacement-related quantities. The TPHATP-based cell showed a stronger directional asymmetry in neutron transmission and reflection, associated with the asymmetric layer sequence and the larger fraction of light elements in the organic cathode and electrolyte-containing components. Despite differences in individual particle yields and spectral characteristics, the TPHATP and LiFePO_4_ models retained broadly similar dominant interaction mechanisms. These results characterize neutron transport and layer-resolved radiation response in the complete cell architecture but do not establish electrochemical radiation tolerance, which requires molecular-scale modelling and experimental validation.

## 1. Introduction

Rechargeable batteries are widely used in portable electronics, electric vehicles, stationary energy-storage systems, and autonomous devices. Their high specific energy and long cycle life also make them attractive for spacecraft, satellites, planetary probes, and other systems that must operate without continuous access to an external power supply [1,2]. Lithium-ion batteries currently dominate many of these applications, while alternative battery chemistries, including potassium-ion systems, are being actively investigated because of their potentially lower cost and the greater natural abundance of potassium [3,4,5,6]. The performance of modern batteries is largely determined by the properties of their electrode materials [7], electrolyte [8], separator [9,10], and interfaces [9].

Battery operation in space is affected by several environmental factors that are less critical under terrestrial conditions. These include vacuum, large temperature variations [5,11], repeated thermal cycling [11], mechanical loads during launch [12], and exposure to ionizing radiation [1,13]. Low temperatures can substantially reduce ionic mobility and charge-transfer rates, whereas elevated temperatures may accelerate electrolyte decomposition, interfacial degradation, and other ageing processes [1,11]. Radiation introduces an additional source of structural and chemical changes that may accumulate during long-duration missions [13,14,15].

The radiation environment of spacecraft includes energetic electrons, protons, heavy ions, γ-rays, and neutrons [16,17,18,19]. In contrast to charged particles, neutrons do not lose energy through direct Coulomb interactions with electrons [20]. They can therefore penetrate deeply into a battery cell and interact with both external components and internal functional layers. Depending on their energy and the elemental composition of the target, neutrons may undergo elastic and inelastic scattering, radiative capture, and other nuclear reactions [20,21,22,23]. These interactions may generate recoil atoms, secondary charged particles, photons, and radioactive nuclei [17,22]. The resulting energy deposition and atomic displacements can potentially modify electrode materials, current collectors, separators, and electrode/electrolyte interfaces.

Elastic neutron scattering is particularly effective for light nuclei because a large fraction of the neutron energy can be transferred in a single collision [22]. This aspect is relevant to current battery-development trends, which increasingly involve organic electrodes, polymeric binders, carbon-based additives, composite separators, and other materials rich in light elements. At the same time, battery cells also contain heavier elements, for which neutron-induced nuclear reactions and secondary-particle production may become important [17,20,22]. A realistic assessment therefore requires the complete multilayer cell to be considered rather than an isolated active material.

The radiation response of lithium-ion and solid-state batteries has previously been studied experimentally [5,10,24,25] and computationally [15]. More broadly, computational approaches are increasingly used to investigate battery materials at different scales, including polymer/coating interfaces [26], electrode-manufacturing and force-field parametrization [27], and structural properties of alkali and transition-metal systems [28]. In particular, recent Geant4 calculations for a solid-state lithium-ion cell considered neutron- and γ-induced non-ionizing energy loss, primary knock-on atoms, secondary nuclear products, induced radioactivity, and radiation-induced current in inorganic Li, LiFePO_4_, and oxide solid-electrolyte components [15]. These studies demonstrate the usefulness of Monte Carlo transport methods for resolving radiation interactions within individual battery materials and layers. However, the available computational literature has predominantly focused on lithium-based systems and inorganic active materials. To the best of our knowledge, neutron transport in a potassium-ion cell containing a molecular organic cathode has not been investigated using a layer-resolved Monte Carlo approach, and direct organic–inorganic cathode comparisons under an identical cell geometry remain lacking. In particular, the combined effects of cathode chemistry on neutron transmission and reflection, layer-resolved energy transfer, secondary particle production, and displacement-related quantities have not been systematically compared within the same multilayer cell model.

In this study, we use Monte Carlo simulations to examine neutron transport and radiation-induced effects in a potassium-ion coin cell with a TPHATP-based cathode [29]. A geometrically equivalent cell with a LiFePO_4_-based cathode was introduced as a physically distinct inorganic reference for cathode substitution comparison rather than as a composition-controlled analogue of the TPHATP electrode. The comparison was used to determine whether cathode composition modifies neutron transmission, reflection, layer-resolved energy transfer, secondary particle production, and displacement-related response within the complete asymmetric cell assembly.

## 2. Calculation Details

### 2.1. Monte Carlo Model

Neutron transport was simulated by using Geant4 version 11.3.2 [30,31,32]. The physics list included high-precision neutron elastic scattering, the G4HadronPhysicsFTFP_BERT_HP hadronic inelastic constructor [33,34,35], elastic and inelastic interactions [34], stopping processes [32], gamma-nuclear interactions [36], electromagnetic interactions described by G4EmStandardPhysics [37], particle decay [32,38], and radioactive decay [39]. Energy-dependent neutron interaction cross sections were obtained from the G4NDL 4.7.1 [40] evaluated nuclear data library.

For a material composed of several elements, the energy-dependent macroscopic cross section was determined as

(1) $$\Sigma \left(E\right)=\sum_{i}n_{i}\sigma_{i}\left(E\right),$$

where $n_{i}$ is the atomic number density of element i, and $\sigma_{i}\left(E\right)$ is the corresponding microscopic interaction cross section at neutron energy E. The probability that a neutron undergoes at least one interaction while traversing a path length l in the material is

(2) $$P\left(E,\;l\right)=1-\exp\left[-\Sigma \left(E\right)l\right].$$

In Geant4, the interaction type and free path are sampled using the energy-dependent cross sections provided by the evaluated neutron data library and the local material composition.

Atomic de-excitation processes [33,37,41], including fluorescence, Auger electron emission, and particle-induced X-ray emission, were enabled. Default Geant4 production cuts, as defined by SetCutsWithDefault() in the physics list, were used for all particle types, with no region-specific production cuts introduced. The lower energy limit of the electromagnetic models was set to 10 eV, the higher energy limit was 100 MeV.

### 2.2. Neutron Source

The incident particles were prompt fission neutrons from thermal neutron-induced fission of ^235^U. In spacecraft environments, secondary neutrons are produced when galactic cosmic rays, solar energetic particles, and trapped particles interact with the spacecraft structure and surrounding matter. Their energies extend from thermal and epithermal values to hundreds of MeV and, in principle, to much higher energies. However, the neutron population relevant to transport and energy deposition inside shielded spacecraft is commonly concentrated in the sub-MeV to several tens of MeV range, whereas the fluence decreases substantially toward higher energies. Measurements and transport analyses for crewed spacecraft have therefore often focused on neutron energies below approximately 15–20 MeV, while also recognizing the presence of a less abundant high-energy component extending beyond 100 MeV [42,43]. Three evaluated prompt-fission neutron spectra were imported from ENDF/B-VIII.0 [44], JEFF-3.3 [45], and JENDL-5 [46]. The corresponding probability density functions were at an incident neutron energy of 0.0253 eV.

These spectra used here are therefore not intended to reproduce a specific orbital neutron environment. Instead, they provide well-defined, evaluated neutron source distributions for comparative analysis of transport and layer-resolved response within the cell.

The tabulated spectra were normalized by piecewise-linear integration. Primary neutron energies were then sampled by analytical inversion of the linear probability density segments. The mean neutron energies were approximately 2.00 MeV for all three evaluations, although the detailed shapes and upper energy limits of the distributions differed. The three evaluated spectra have similar mean energies but differ slightly in their detailed shapes and upper energy limits. Their probability density functions are compared in Figure S1 of the Supplementary Information (SI).

A parallel circular neutron beam with a radius of 9.5 mm was used. The source was placed 10 mm from the upstream surface of the cell and was directed along the axis normal to the battery layers. Two irradiation geometries were considered—bottom-to-top and top-to-bottom. Each combination of source spectrum and irradiation direction was simulated using 10^7^ primary neutrons.

In addition to the prompt fission neutron source-driven (PFNS-driven) calculations, illustrative monoenergetic simulations were performed at 2 and 30 MeV in order to examine the energy distributions of secondary particles produced in the cell. These auxiliary calculations used 10,000 incident neutrons.

### 2.3. Battery Geometry and Materials

**Figure 1:**
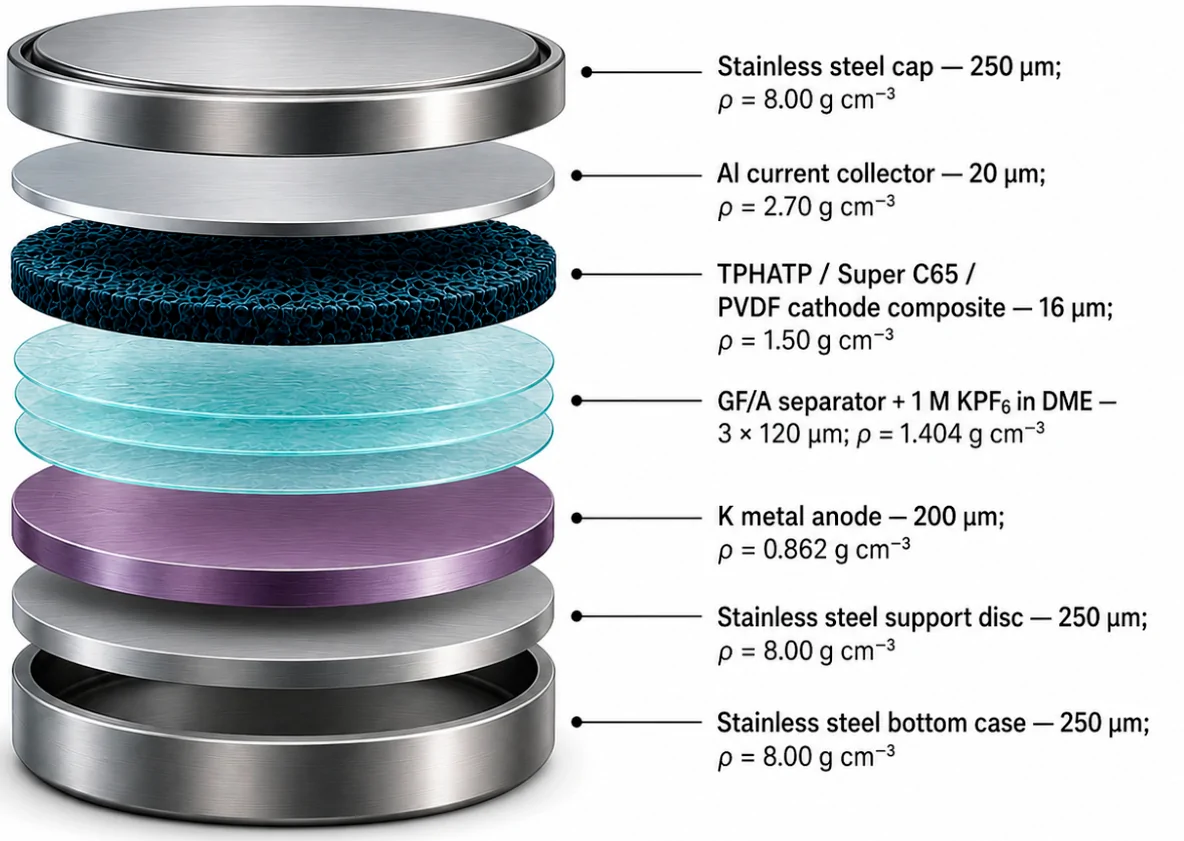
Geant4 model of the CR2032 potassium-ion cell containing a composite TPHATP cathode.

The simulated geometry represented a CR2032 potassium-ion coin cell. The cell was modelled as a stack of coaxial cylindrical layers, as illustrated in Figure 1 (this figure was generated using AI instruments). Its main components were two stainless steel case elements, a stainless-steel support disc, a metallic potassium anode, three electrolyte-impregnated glass-microfiber separator layers, a composite hydrated TPHATP (triphenazino[2,3-b](1,4,5,8,9,12-hexaazatriphenylene), C_42_H_18_N_12_·6H_2_O) cathode, and a carbon-coated aluminium current collector.

The experimental cell architecture was taken from the work [29]. The reported composite cathode contained 50 wt.% TPHATP, 40 wt.% Timical Super C65 conductive carbon, and 10 wt.% poly(vinylidene fluoride) (PVDF). The cathode was deposited onto carbon-coated aluminium foil. Metallic potassium pressed onto a stainless steel disc was used as the counter electrode. Three Whatman GF/A glass-microfiber filters served as separators, and 80 μL of 1 M KPF_6_ in dimethoxyethane was added to each CR2032 cell. A complete summary of the Geant4 material definitions, including elemental or composite compositions, densities, porosities, and effective medium assumptions for heterogeneous components, is provided in Table S1 of SI.

Because the original experimental report did not provide the assembled thickness and diameter of every internal component, several geometric parameters had to be introduced as modelling assumptions. The baseline geometry used an effective total stack thickness of 1.346 mm. The cathode thickness was calculated as 0.016 mm from the reported active-material loading, cathode composition, and assumed composite density. Each separator was represented as a 0.120 mm thick homogeneous effective medium containing a glass-microfiber matrix and electrolyte-filled porosity.

The assumed dimensions were applied identically in all baseline simulations. They were therefore used to compare neutron spectra and irradiation directions rather than to reproduce the exact response of a specific manufactured coin cell.

Additional calculations involving a reference LiFePO_4_ cathode and enlarged TPHATP and LiFePO_4_ cathode layers were performed as supplementary sensitivity studies.

These calculations were used to examine the influence of cathode composition and thickness on interaction statistics and the spatial localization of neutron-induced processes. The corresponding results are presented in Supplementary Information (SI).

### 2.4. Recorded Quantities and Data Normalization

During each simulation, the code recorded neutron transport through the multilayer structure, energy deposition in individual cell components, interaction processes, secondary-particle production, recoil nuclei, and neutron crossings of the external cell boundaries. Separate spectra were accumulated for incident, transmitted, and reflected neutrons.

Primary knock-on atoms (PKA) were recorded for subsequent displacement damage calculations. Non-ionizing energy deposition, Lindhard [47] damage energy, and Norgett–Robinson–Torrens (NRT)-equivalent displacements per atom (DPA) [48] values were treated as distinct quantities and were not used interchangeably. Spectrum-averaged NRT displacement cross sections were calculated for selected solid materials using specified threshold displacement energies. For potassium, the threshold displacement energy was treated parametrically because a validated value was unavailable.

Since no absolute neutron fluence was specified, deposited energies, particle yields, and process counts are reported per primary neutron. The displacement results are therefore presented as spectrum averaged cross sections rather than as absolute values of DPA.

This normalization was intentionally retained to permit direct comparison between the simulated cell configurations without introducing mission-specific assumptions. Conversion of these quantities to accumulated deposited energy or DPA would require a neutron fluence and spectral distribution defined for a particular orbit, shielding configuration, spacecraft geometry, and mission duration.

Spatial event distribution maps for the TPHATP and LiFePO_4_ cathode models with layer thicknesses of 16 and 50 um are presented in Figures S2 and S3, respectively. The maps were constructed for monoenergetic neutron irradiation at 2 MeV, close to the mean PFNS energy, and 30 MeV, representative of the high energy end of the evaluated PFNS range and were used for qualitative visualization of the spatial localization of primary neutron steps and secondary particle events.

## 3. Results and Discussion

The simulated response of the TPHATP-based cell was analyzed in terms of neutron transport, energy deposition, secondary-particle production, interaction processes, and displacement-related quantities. The effects of the incident prompt fission neutron spectrum and irradiation direction were considered separately, since the cell has an asymmetric multilayer structure. We first examine the transmitted and reflected neutron components to establish how the cell modifies the incident spectra. The layer-resolved energy deposition, particle yields, interaction processes, and displacement response are discussed subsequently.

### 3.1. Neutron Transmission and Reflection by the TPHATP-Based Cell

**Figure 2:**
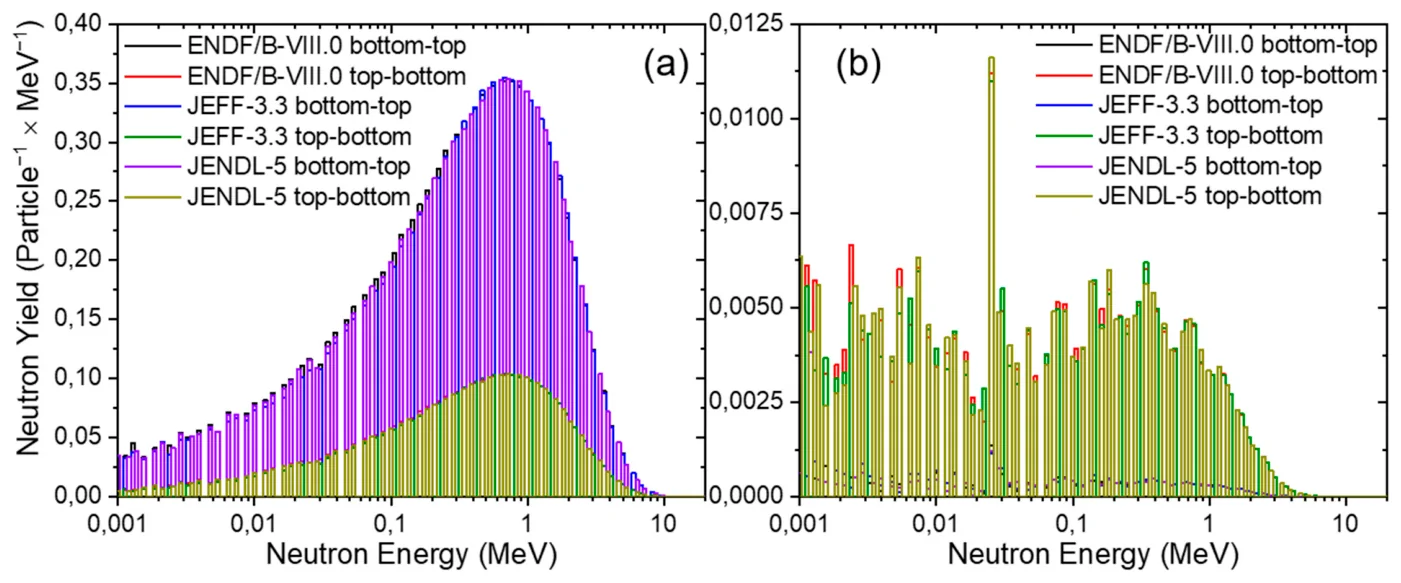
Neutron energy spectra calculated for the TPHATP-based CR2032 cell: (**a**) transmitted neutron spectra and (**b**) reflected neutron spectra. Results are shown for the three neutron spectra and both irradiation directions.

Figure 2 presents the differential energy distributions of neutrons crossing the downstream and upstream boundaries of the cell. The results are compared for the ENDF/B-VIII.0, JEFF-3.3, and JENDL-5 source spectra and for both irradiation directions.

The transmitted spectra (Figure 2a) largely preserve the overall shape of the incident neutron distributions, indicating that substantial fraction of neutrons traverse the thin CR2032 stack without a large change in energy. Nevertheless, the transmitted yields and spectral shapes depend on the irradiation direction because the cell has an asymmetric multilayer structure. In particular, the lower transmitted yield observed for top-to-bottom irradiation is accompanied by a higher reflected yield (Figure 2b).

This directional redistribution may partly reflect the order in which neutrons encounter the hydrogen-containing organic cathode and electrolyte-rich separator layers, where elastic scattering from light nuclei can promote neutron moderation and increase the likelihood of return crossings at the upstream boundary. However, the directional response represents the combined effect of the entire layer sequence and cannot be attributed to the cathode alone.

**Figure 3:**
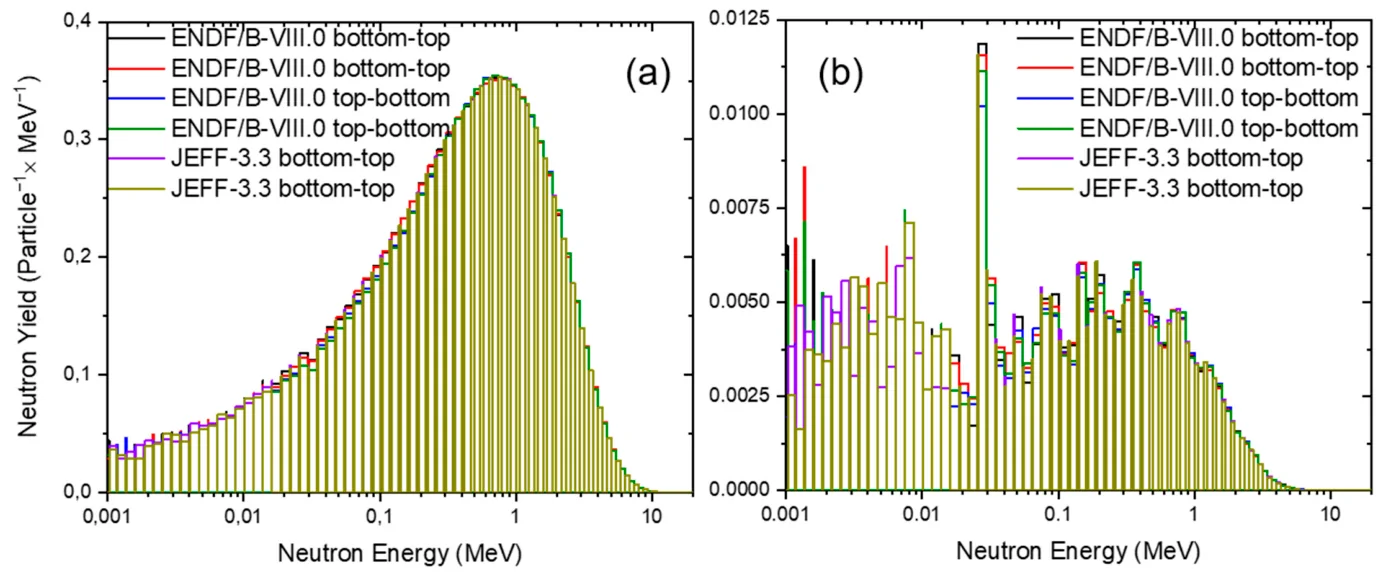
Neutron energy spectra calculated for the LiFePO_4_-based CR2032 cell: (**a**) transmitted neutron spectra and (**b**) reflected neutron spectra. Results are shown for the three evaluated ^235^U prompt fission neutron spectra and both irradiation directions.

To assess whether the observed directional response was specific to the TPHATP-containing cathode, an otherwise identical reference cell containing a LiFePO_4_-based cathode was also considered (Figure 3).

In contrast to the TPHATP-based cell, the LiFePO_4_ reference model showed only minor differences between the two irradiation directions. The transmitted spectra (Figure 3a) nearly coincided over most of the energy range, and no comparable reduction in the top-to-bottom transmitted yield was observed. The reflected spectra (Figure 3b) likewise showed a weaker directional dependence.

This comparison supports the assumption that the more pronounced asymmetry in the TPHATP-based model is partly associated with the composition and position of the hydrogen-containing cathode, although the response remains influenced by the complete multilayer structure. Accordingly, the differences between the two reference models should be interpreted as the combined consequence of cathode composition, density, and formulation, rather than as an isolated effect of organic versus inorganic character.

The radial exit distributions obtained at 2 and 30 MeV are presented in Figure S4. The transmitted neutron profiles are nearly identical for the two irradiation directions and increase toward larger exit radii because of the larger annular area represented by each radial interval. In contrast, neutrons returning through the irradiated surface are much less numerous and show stronger statistical fluctuations. No pronounced systematic directional difference is evident in the radial profiles.

### 3.2. Layer-Resolved Energy Transfer and Displacement-Related Response

**Figure 4:**
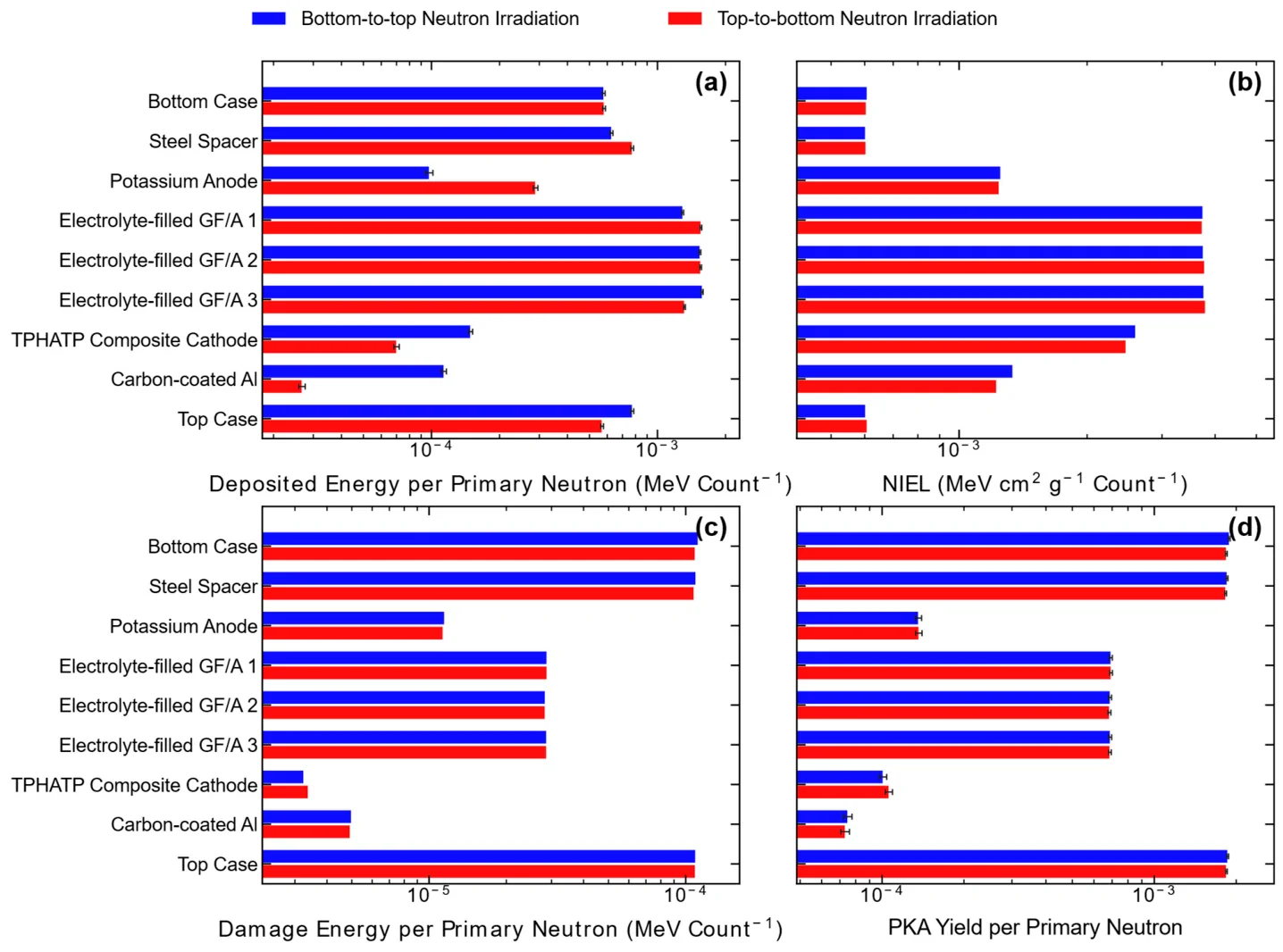
Layer-resolved response of the TPHATP-based CR2032 cell calculated using the ENDF/B-VIII.0 prompt fission neutron spectrum: (**a**) deposited energy per primary neutron, (**b**) NIEL per primary neutron, (**c**) damage energy per primary neutron, and (**d**) PKA yield per primary neutron.

To identify the internal components responsible for energy transfer and recoil production, the response was analyzed separately for each cell layer. Figure 4 compares the deposited energy, non-ionizing energy loss (NIEL), damage energy, and PKA yield for the two irradiation directions.

For each irradiation direction, the deposited energy was normalized to the number of primary neutrons as ${\bar{E}}_{dep}=E_{dep,total}/N_{primary}$. The uncertainty shown in Figure 4a is the standard error of the mean obtained from the event-by-event deposited energy distribution, $SEM=s\left(E_{dep,event}\right)/\sqrt{N_{primary}}$. The PKA yield was calculated as $Y_{PKA}=N_{PKA}/N_{primary}$, and its uncertainty was estimated from Poisson counting statistics as $\sigma \left(Y_{PKA}\right)=\sqrt{N_{PKA}}/N_{primary}$ (Figure 4d). Error bars are not shown for NIEL and damage energy because the event-by-event variances of the corresponding quantities were not retained in the simulation output. Their plotted values therefore represent normalized point estimates derived from the accumulated run totals.

The layer-resolved response is strongly non-uniform across the cell components. The largest deposited energy values are obtained for the electrolyte-containing separator region and the stainless steel case elements, reflecting the combined effects of layer thickness, density, composition, and interaction probability. Since the deposited energy in Figure 4a includes both ionizing and non-ionizing contributions, it represents the total energy transferred locally within each layer. Neutrons do not produce substantial direct ionization because they carry no electric charge. Instead, the ionizing contribution is generated indirectly by secondary charged particles, recoil nuclei, and electrons produced in neutron interactions. These particles subsequently lose energy through excitation and ionization as they propagate through the material.

The relatively thick separator and steel layers show only weak differences between the two irradiation directions. In contrast, the potassium anode, TPHATP cathode, and aluminium collector exhibit a more pronounced directional dependence because neutrons reach these thin components after traversing different preceding layer sequences. Prior scattering and moderation modify the neutron energy distribution entering each layer and may increase the probability of low energy reactions in materials whose cross sections rise as the neutron energy decreases.

The NIEL values are comparatively high in the hydrogen containing organic cathode and electrolyte filled separator layers (Figure 4b). This response is associated with efficient kinetic energy transfer from neutrons to light nuclei during elastic scattering. Because the neutron and proton masses are similar, hydrogen can receive a large fraction of the incident neutron energy in a single collision, while carbon, nitrogen, oxygen, fluorine, and silicon can also produce appreciable recoil energies.

In addition, NIEL is normalized by the layer areal density (ρt), and therefore represents non-ionizing energy transfer per unit mass thickness rather than the total energy accumulated in the layer. The relatively high NIEL values in the cathode and separator layers therefore indicate efficient recoil energy transfer relative to their areal density, rather than a predominance of non-ionizing energy deposition over the total deposited energy. Accordingly, these NIEL values should be interpreted as energy transfer metrics and not as direct predictors of persistent structural damage or electrochemical degradation, particularly in molecular and liquid-containing phases.

To estimate the thermal significance of the deposited energy, an effective layer-averaged adiabatic temperature rise was calculated for the TPHATP-based and LiFePO_4_-based cells under monoenergetic neutron irradiation at 2 and 30 MeV (Figures S10 and S11). The cathodes were treated as solid composite layers, whereas the electrolyte-filled GF/A separators were represented as porous glass-fibre media containing liquid electrolyte. For these heterogeneous components, the calculated temperature rise represents an effective value averaged over the complete layer using its assigned mass and heat capacity. The resulting temperature increments remain on the order of 10^−9^ K per primary neutron, but its layer-resolved distribution identifies which cell components convert the largest fraction of deposited energy into heat. These values should not be interpreted as operational temperature changes of the battery. In the absence of absolute fluence, irradiation time profile, and thermal boundary conditions, they represent only normalized layer-resolved indicators of energy-to-heat conversion under the simulated irradiation conditions. At 2 MeV, the largest values are generally obtained for the electrolyte-filled separator layers and composite cathodes. At 30 MeV, the thermal response becomes more direction-dependent, with comparatively large values in the potassium anode and, for some irradiation geometries, the carbon-coated aluminium current collector. Thus, the calculation provides a comparative measure of the relative energy-to-heat response of individual layers, irradiation directions, neutron energies, and cathode compositions.

Damage energy (Figure 4c) and PKA yield (Figure 4d) are largest in the metallic case elements. These layer-integrated quantities are governed by the amount of material, atomic number density, neutron reaction cross sections, and recoil energy distributions. The steel components provide a comparatively large interaction volume and support multiple elastic and non-elastic reaction channels capable of generating recoil nuclei. Although medium- and high-mass nuclei can produce energetic recoils and displacement cascades, the larger values observed in the metallic layers should not be attributed to atomic mass alone. In particular, the fraction of neutron energy transferred in a single elastic collision decreases with increasing target mass. The high PKA yields therefore primarily reflect the larger integrated probability of nuclear interactions in the thick and dense metallic components, whereas the much thinner cathode and collector layers produce fewer recoil nuclei in absolute terms.

Overall, the layer resolved results show that different cell components dominate different response quantities. Total energy deposition is largest in the separator region and steel components because of their greater thickness, material content, and integrated interaction probability. NIEL is enhanced in layers containing light elements, particularly the electrolyte filled separators and the TPHATP composite cathode, because elastic scattering transfers energy efficiently to hydrogen and other light nuclei. Damage energy and PKA production are largest in the steel case elements because their thickness and atomic number density provide a greater integrated probability of recoil producing nuclear interactions. The corresponding results calculated using the JEFF-3.3 and JENDL-5 prompt fission neutron spectra, together with the layer-resolved results for the LiFePO_4_ reference cell, are presented in Figures S5–S9.

### 3.3. Secondary Particles and Interaction Processes

**Figure 5:**
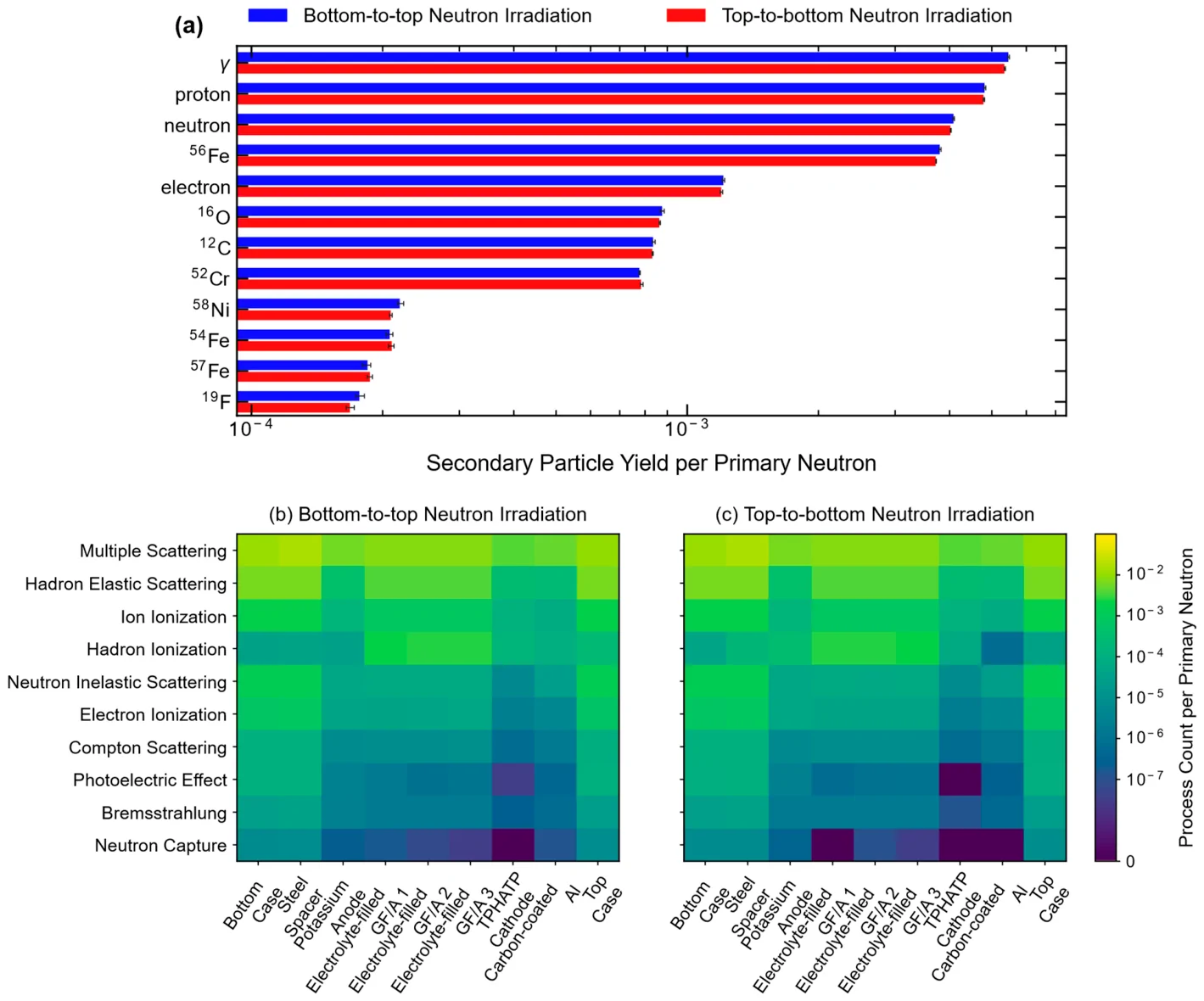
Secondary particle production and interaction process distribution in the TPHATP-based CR2032 cell: (**a**) secondary particle yield per primary neutron for the most abundant particle and recoil species under bottom-to-top and top-to-bottom irradiation; (**b**) layer-resolved process counts per primary neutron for bottom-to-top irradiation; and (**c**) layer-resolved process counts per primary neutron for top-to-bottom irradiation.

To further clarify how the neutron response of the cell is formed, the production of secondary particles and the distribution of interaction processes were analyzed. Figure 5 summarizes the yields of the most abundant secondary species, and the layer-resolved process counts for the two irradiation directions.

Figure 5a shows that the most abundant secondary products are γ-quanta, protons, neutrons, and recoil nuclei originating mainly from the metallic cell components, such as Fe-isotopes, ^52^Cr, and ^58^Ni. Electrons and light-element recoils, including ^16^O and ^12^C, also make a noticeable contribution. The yields of the dominant secondary species are broadly similar for the two irradiation directions, indicating that the overall level of neutron-induced secondary particle production is only weakly affected by reversing the stack orientation. However, small but systematic differences remain for several species, reflecting the influence of the asymmetric layer sequence on the local neutron spectrum and on the relative probabilities of scattering, capture, and non-elastic nuclear reactions.

The process maps in Figure 5b,c show where the recorded transport and interaction processes occur within the cell. Multiple scattering and hadron elastic scattering provide the largest counts across most layers. Ionization processes are also frequently recorded, especially in the electrolyte-filled separators and in the metallic components. These ionization events do not represent direct ionization by primary neutrons. They are produced by recoil nuclei, electrons, protons, and other charged secondary particles generated in neutron interactions. Therefore, the high ionization process counts are consistent with the deposited energy results and do not contradict the NIEL analysis, which accounts only for the non-ionizing part of the transferred energy.

Neutron capture, photoelectric absorption, and other less frequent processes show lower and more localized counts.

The overall process distributions shown in Figure 5b,c are very similar for the two irradiation directions. No pronounced systematic differences can be identified from the process maps, indicating that reversal of the cell orientation does not substantially change the dominant interaction mechanisms. The directional effects observed in the transmitted and reflected neutron spectra (Figure 2) and in the layer-resolved deposited energy (Figure 4a) are therefore more apparent in the integrated quantitative results than in the qualitative process maps.

The PFNS-based calculations characterize the integrated secondary particle response over the broad fission neutron energy distribution. Since reversing the irradiation direction produced only minor changes in the total yields and relative abundances of the dominant secondary species (Figure 5a), the energy-resolved analysis was restricted to a single irradiation direction.

**Figure 6:**
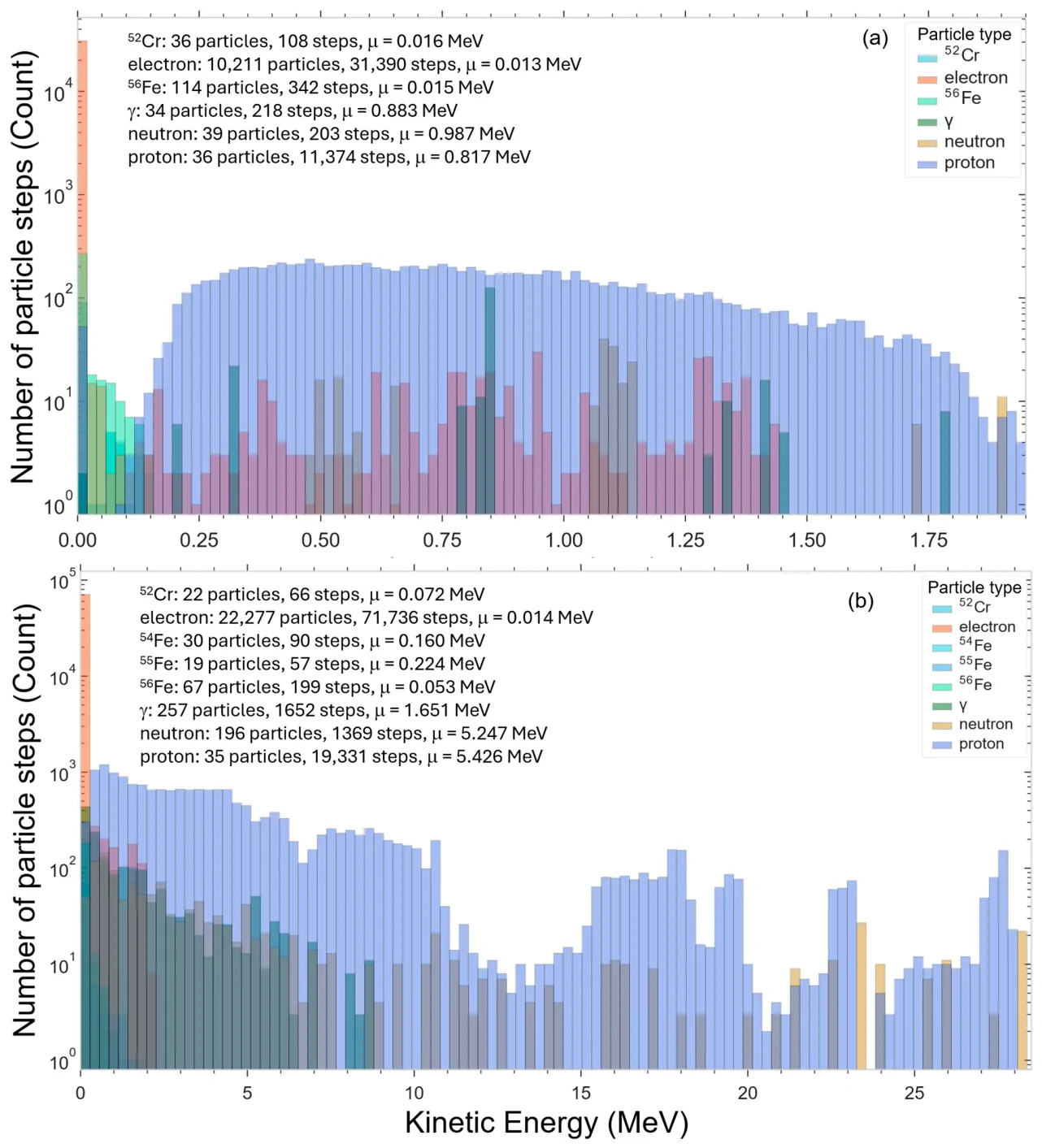
Energy distributions of secondary particles produced in the TPHATP-based CR2032 cell under monoenergetic neutron irradiation at (**a**) 2 MeV and (**b**) 30 MeV. The annotations report the number of recorded particles, the number of transport steps, and the mean kinetic energy μ for each selected species.

To examine how the secondary particle spectra change with incident neutron energy, additional illustrative simulations were performed for monoenergetic 2 and 30 MeV neutrons (Figure 6). Each case used 10^4^ primary neutrons and was intended for qualitative mechanistic comparison rather than high-precision yield estimation.

At 2 MeV, the secondary field is dominated by low energy electrons and recoil protons, while only limited numbers of γ-quanta, secondary neutrons, and heavy recoil nuclei are produced. The proton spectrum extends across a substantial fraction of the incident energy range, consistent with efficient elastic energy transfer to hydrogen-containing components of the cell. At 30 MeV, the secondary spectra become broader and more energetic, and the contributions from secondary neutrons, γ-quanta, and iron isotopes become more pronounced. This behaviour is consistent with the increasing importance of non-elastic nuclear reaction channels at higher incident neutron energy.

The extended secondary particle distributions show that both cathode models produce broadly similar dominant species, including photons, protons, secondary neutrons, electrons, and recoil nuclei originating mainly from the steel components. The LiFePO_4_ reference cell does not show a qualitatively different interaction pattern; multiple scattering, hadron elastic scattering, and ionization by secondary charged particles remain the dominant recorded processes. Differences between the two models are mainly associated with less abundant recoil species and with the local process counts in the cathode layer, reflecting the different elemental compositions of TPHATP and LiFePO_4_ rather than a change in the overall response mechanism. More detailed secondary particle yields and process-resolved maps for both cells are presented in Figures S12–S15.

The corresponding monoenergetic results for the LiFePO_4_ reference cell are shown in Figure S16. The LiFePO_4_-based model exhibits the same general energy dependence as the TPHATP-based cell. At 2 MeV, secondary-particle production is dominated by low-energy electrons and recoil protons, whereas photons, secondary neutrons, and heavy recoil nuclei remain comparatively scarce. At 30 MeV, the spectra broaden, the mean energies of the recorded species increase, and secondary neutrons, photons, and heavy recoil products become more prominent because additional non-elastic reaction channels are accessible.

Comparison of Figure 6 and Figure S16 shows that the TPHATP-based and LiFePO_4_ cells produce secondary particle spectra of the same general scale, although the distributions are not identical. At 2 MeV, the numbers of comparable secondary species are generally of the same order of magnitude, and their mean kinetic energies are similar.

However, the maximum energies and the yields of individual recoil products can differ between the two cathode models. The LiFePO_4_ results also include a somewhat broader set of low-yield reaction products.

The slightly larger secondary particle yields obtained for the TPHATP-based cell can be related to its higher content of light elements, particularly hydrogen, carbon, and nitrogen, which promotes efficient neutron energy transfer and the production of light charged secondaries.

These particles subsequently generate additional electrons and photons, while at 30 MeV the broader reaction cascade also increases secondary neutron production. The resulting differences remain modest because most secondary particles are produced throughout the complete multilayer cell rather than exclusively within the cathode.

### 3.4. Spectrum-Averaged Displacement Cross Sections

The secondary particle and interaction process analyses describe the mechanisms of energy transfer within the cell, but they do not directly quantify the resulting displacement response of the constituent materials. Therefore, spectrum-averaged NRT displacement cross sections were calculated for selected metallic components using the corresponding threshold displacement energies [27,28,49,50,51,52,53,54]. The threshold displacement energies used in the calculations, together with their literature sources and physical context, are summarized in Table S2. These values should be regarded as material- and phase-specific modelling parameters rather than universal elemental constants.

No values were reported for the liquid electrolyte because a fluid has no persistent lattice sites and the concept of a stable atomic displacement is therefore not directly applicable. Moreover, for the molecular TPHATP phase, conventional NRT displacements would not directly capture the dominant radiation-induced chemical changes, including bond scission, radical formation, and molecular fragmentation. These components were therefore characterized by using deposited energy, NIEL, damage energy, and secondary-particle metrics rather than NRT displacement cross sections. Accordingly, the NRT-based analysis presented below should be interpreted primarily as a displacement-response description of the metallic cell components rather than as a direct measure of radiation-induced degradation in the molecular cathode or liquid electrolyte.

**Figure 7:**
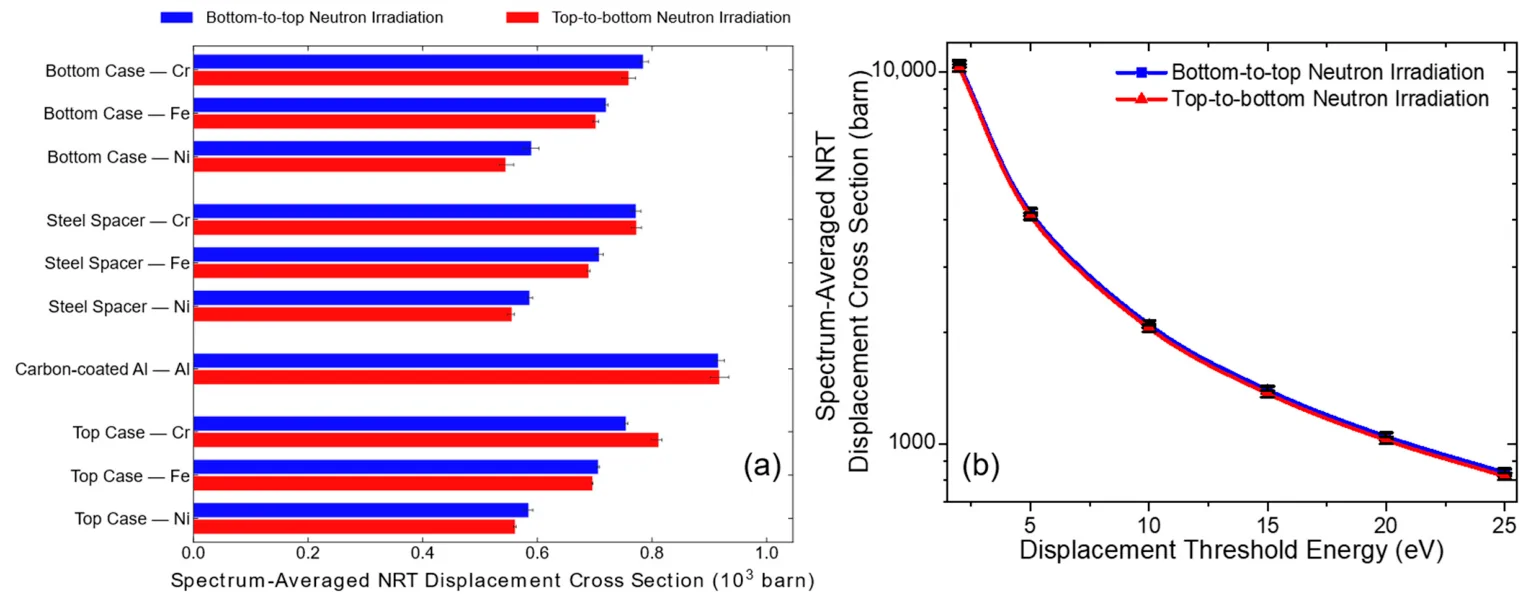
Spectrum-averaged NRT displacement response of selected cell materials: (**a**) displacement cross sections for Cr, Fe, Ni, and Al in the metallic cell components, (**b**) dependence of the spectrum-averaged displacement cross section of metallic potassium on the assumed threshold displacement energy under bottom-to-top and top-to-bottom irradiation.

Figure 7 compares the spectrum-averaged NRT displacement cross sections for the principal metallic elements in the cell under the two irradiation directions. The sensitivity of the potassium result to the assumed threshold displacement energy is shown separately because no validated $E_{d}$ value is available for metallic potassium.

The spectrum-averaged displacement cross sections are of the order of $10^{3}$ barn and vary moderately among the metallic elements and cell components (Figure 7a). Chromium generally exhibits the largest values, followed by iron and nickel, whereas aluminium shows a similarly high response in the current collector.

These differences reflect the combined effects of the elemental recoil spectra, neutron-reaction cross sections, atomic masses, and adopted threshold displacement energies rather than atomic mass alone. The broader variability and material dependence of threshold displacement energies in crystalline solids, including ceramics and oxides, have been discussed extensively in previous reviews [55,56]. Reported $E_{d}$ values may vary with crystal structure, crystallographic direction, temperature, and the experimental or computational method used for their determination. Consequently, the relative displacement response should be interpreted within the specific $E_{d}$ assumptions adopted here.

The two irradiation directions produce closely similar displacement cross sections for most components. The remaining differences are associated with the asymmetric layer sequence and the resulting changes in the neutron spectrum reaching each material.

The largest directional differences occur in components located near the outer regions of the stack, whereas the steel spacer and aluminium collector show comparatively similar values for the two geometries.

The calculated potassium displacement cross section decreases monotonically as the assumed threshold displacement energy increases (Figure 7b). This trend follows directly from the NRT formalism—a higher $E_{d}$ reduces the fraction of recoil events capable of producing stable atomic displacements and lowers the estimated number of displacements per recoil. The two irradiation directions yield nearly overlapping dependences, indicating that uncertainty in the potassium threshold displacement energy has a substantially greater influence on the calculated displacement response than the orientation of the cell.

Because no absolute neutron fluence was specified, these results should not be interpreted as absolute DPA values. They represent spectrum-averaged displacement cross sections that can subsequently be converted to DPA for a specified irradiation fluence.

Overall, the displacement response is comparatively insensitive to irradiation direction but remains dependent on material-specific displacement parameters. In particular, the assumed threshold displacement energy for metallic potassium has a much stronger effect on the predicted NRT response than the irradiation direction. The corresponding supplementary results are provided for the LiFePO_4_ reference cell (Figure S17).

## 4. Conclusions

A multilayer Geant4 model of a TPHATP-based CR2032 potassium-ion cell was developed for irradiation by prompt fission neutron spectra. The results show that the neutron response is governed by the complete asymmetric cell architecture rather than by the cathode alone. The hydrogen-containing internal layers affect moderation and energy transfer, while the steel case and spacer dominate recoil and secondary isotope production, including Fe-, Cr-, and Ni-containing products. The TPHATP-based cell exhibits a more pronounced directional asymmetry in neutron transmission and reflection than the LiFePO_4_ reference model, indicating that both cathode composition and its position within the layer sequence influence the transport response. The displacement analysis is most reliable for the metallic components, whereas the NRT response of potassium is controlled mainly by the assumed threshold displacement energy. For the electrolyte, carbon phases, and molecular TPHATP, lattice displacement metrics alone are insufficient to describe radiation damage. Accordingly, the present results should be interpreted as a comparative neutron-transport and radiation-response analysis rather than as a demonstration of electrochemical radiation tolerance or space qualification of the investigated cell chemistry.

The model includes several limitations. Dimensions of some internal components were assumed, and porous or composite layers were represented as homogeneous effective media. All results were normalized per primary neutron because no absolute fluence was specified, while the 2 and 30 MeV monoenergetic calculations were used only for qualitative mechanistic interpretation. The prompt fission neutron spectra used in this work should be regarded as model irradiation fields rather than as direct representations of mission-specific spacecraft neutron spectra. Accordingly, the reported quantities should be interpreted as normalized comparative radiation-response metrics rather than as mission-integrated engineering estimates. The model does not account for electrochemical operation, irradiation-induced chemical reactions, structural relaxation, or subsequent changes in cycling performance. Future work should therefore combine neutron transport calculations with molecular dynamics and DFT methods to relate local energy transfer and recoil production to bond breaking, radical formation, and molecular degradation in the cathode and electrolyte, together with neutron-irradiation experiments assessing capacity retention, impedance evolution, and post-irradiation chemical and structural changes.

## Data Availability

The data presented in this study are available from the corresponding author upon reasonable request.

## Supplementary Materials

The following supporting information can be downloaded at https://www.mdpi.com/article/10.3390/nano16181188/s1, Figure S1: Evaluated prompt fission neutron probability density functions for thermal neutron-induced fission of ^235^U obtained from ENDF/B-VIII.0, JEFF-3.3, and JENDL-5; Table S1: Geant4 material definitions and composition of the simulated CR2032 cell components; Table S2: Threshold displacement energies used in the NRT calculations and their physical basis; Figure S2: Event distribution maps in the TPHATP layer of different thickness under 2 and 30 MeV neutron irradiation; Figure S3: Event distribution maps in the LiFePO_4_ layer of different thickness under 2 and 30 MeV neutron irradiation; Figure S4: Radial distributions of neutron tracks exiting the TPHATP-based and LiFePO_4_-based cells under monoenergetic irradiation at 2 and 30 MeV; Figure S5: Layer-resolved response of the TPHATP-based CR2032 cell calculated using the JEFF-3.3 prompt fission neutron spectrum; Figure S6: Layer-resolved response of the TPHATP-based CR2032 cell calculated using the JENDL-5 prompt fission neutron spectrum; Figure S7: Layer-resolved response of the LiFePO_4_-based CR2032 cell calculated using the ENDF/B-VIII.0 prompt fission neutron spectrum; Figure S8: Layer-resolved response of the LiFePO_4_-based CR2032 cell calculated using the JEFF-3.3 prompt fission neutron spectrum; Figure S9: Layer-resolved response of the LiFePO_4_-based CR2032 cell calculated using the JENDL-5 prompt fission neutron spectrum; Figure S10: Normalized adiabatic temperature rise in TPHATP-based cell layers caused by generated secondary particles; Figure S11: Normalized adiabatic temperature rise in LFP-based cell layers caused by generated secondary particles; Figure S12: Secondary particle production in the TPHATP-based CR2032 cell under bottom-to-top and top-to-bottom irradiation; Figure S13: Interaction process distribution in the TPHATP-based CR2032 cell under bottom-to-top and top-to-bottom irradiation; Figure S14: Secondary particle production in the LFP-based CR2032 cell under bottom-to-top and top-to-bottom irradiation; Figure S15: Interaction process distribution in the LFP-based CR2032 cell under bottom-to-top and top-to-bottom irradiation; Figure S16: Energy distributions of secondary particles produced in the LFP-based CR2032 cell under monoenergetic neutron irradiation at 2 and 30 MeV; Figure S17: Spectrum-averaged NRT displacement response of selected cell materials in the LFP-based battery.
