# Supplementary material for: Neutron Transport and Layer-Resolved Radiation Effects in Potassium-Ion Cells with Organic and Inorganic Cathodes Under Space-Relevant Irradiation
Source: Nanomaterials (Basel). 2026 Sep 20;16(18):1188. doi: 10.3390/nano16181188 (PMC13610212; doi:10.3390/nano16181188)
Supplement: Supplementary file 1 [file nanomaterials-16-01188-s001.zip › nanomaterials-4530945-supplementary.pdf]

Neutron Transport and Layer-Resolved Radiation Effects in Potassium-Ion Cells with Organic and Inorganic Cathodes Under Space-Relevant Irradiation

Ivan E. Novoselov [1,2,*] and Ivan S. Zhidkov [1,2,3,*]

[1] Institute of Physics and Technology, Ural Federal University, Mira 19 Street, Yekaterinburg 620062, Russia
[2] Federal Research Center of Problems of Chemical Physics and Medicinal Chemistry of the Russian Academy of Sciences, Semenov Av., 1, Chernogolovka 142432, Russia
[3] M.N. Mikheev Institute of Metal Physics, ral Branch of the Russian Academy of Sciences, S. Kovalevskoi 18 Street, Yekaterinburg 620108, Russia
* Correspondence: i.e.novoselov@urfu.ru (I.E.N.); i.s.zhidkov@urfu.ru (I.S.Z.)

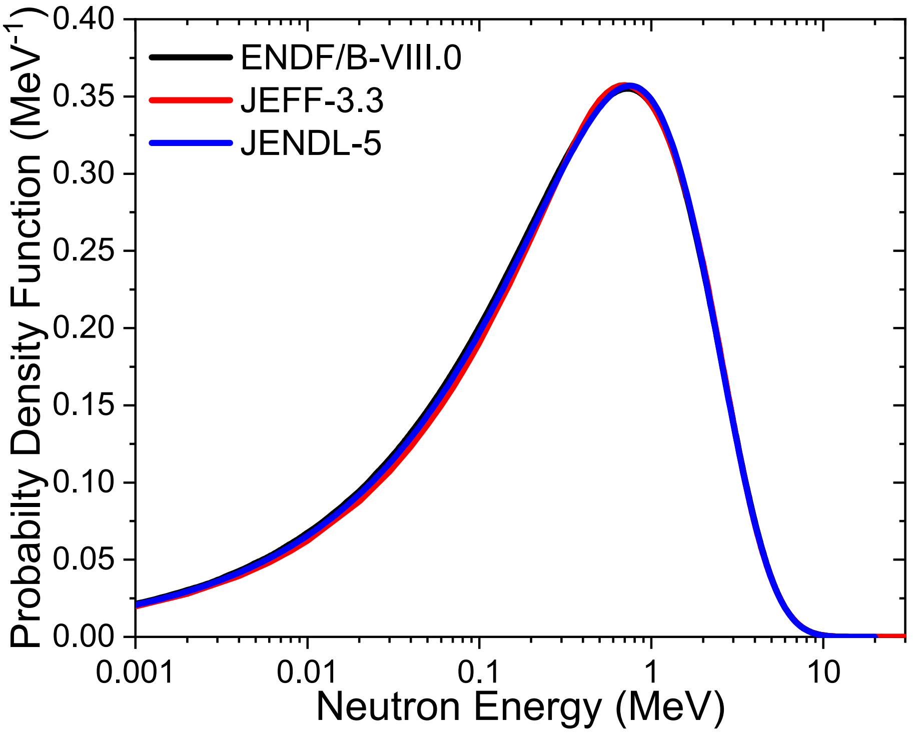

Figure S1. Evaluated prompt fission neutron probability density functions for thermal neutron-induced fission of ^{235}U obtained from ENDF/B-VIII.0, JEFF-3.3, and JENDL-5

For clarity, only representative projections are shown in Figs. S2 and S3. The selected XY projections correspond to regions with the highest densities of particle steps and recorded interaction events. Each simulation comprised 10^4 primary neutrons, since larger runs produced prohibitively large log files for trajectory-level visualization and processing. The selection of projections and the reduced number of primary particles were used only for qualitative illustration and did not affect the quantitative results reported in the main text.

Table S1. Geant4 material definitions and composition of the simulated CR2032 cell components.

Cell component	Composition used in the model	Density / porosity	Modelling note
Bottom stainless steel case	NIST stainless steel composition	NIST database density	Thickness 0.25 mm.
Stainless steel spacer	NIST stainless steel composition	NIST database density	Thickness 0.25 mm.
Potassium anode	K	NIST database density	Metallic potassium; thickness 0.20 mm.
GF/A separators	Pyrex-glass approximation + 1 M KPF_6/DME	Porosity = 0.70; effective density calculated from glass/electrolyte volume fractions	Three separator layers, each 0.12 mm thick.
TPHATP	$C_{42}H_{18}N_{12}\cdot 6H_2O$ ($C_{42}H_{30}N_{12}O_6$)	1.50 g cm^{-3}	Hydrated TPHATP
Super C65	C	2.00 g cm^{-3}	Elemental carbon
PVDF	$(C_2H_2F_2)n$	1.78 g cm^{-3}	Binder in TPHATP composite.
TPHATP composite cathode	50 wt.% TPHATP + 40 wt.% Super C65 + 10 wt.% PVDF	Bulk density = 1.50 g cm^{-3}; porosity = 0.20	Active loading 1.2 mg cm^{-2}; homogeneous composite representation.
LiFePO4	$LiFePO_4$	3.60 g cm^{-3}	Fully lithiated olivine $LiFePO_4$ (LFP).
PAA	$(C_3H_4O_2)n$	1.22 g cm^{-3}	Binder in LFP composite.
$LiFePO_4$ composite cathode	85 wt.% $LiFePO_4$ + 10 wt.% Super C65 + 5 wt.% PAA	Bulk density = 1.50 g cm^{-3}; porosity = 0.20	Density 1.50 g cm^{-3} in the reference/sensitivity model.
Electrolyte	KPF_6 in $C_4H_{10}O_2$	1.047813 g cm^{-3} at 20 °C.	Modelled estimation
Carbon-coated Al current collector	Al	NIST database density	Carbon coating was not represented as a separate layer; foil thickness 0.02 mm.
Top stainless steel case	NIST stainless-steel composition	NIST database density	Thickness 0.25 mm.

Table S2. Threshold displacement energies used in the NRT calculations and their physical basis.

**Material / element**	E_d **used, eV**	**Treatment**	**Physical context / source**
Fe in stainless steel	~28	Nominal	AISI-304-like Fe–Cr–Ni alloy, MD-based
Cr in stainless steel	~28	Nominal	AISI-304-like Fe–Cr–Ni alloy, MD-based
Ni in stainless steel	~28	Nominal	AISI-304-like Fe–Cr–Ni alloy, MD-based
Al	25	Nominal	fcc Al, engineering/MD literature
Al	16 ± 3; 13–14	Literature comparison	Alternative reported ranges, not baseline
K	2, 5, 10, 15, 20, 25	Parametric	No validated E_d identified for metallic bcc K

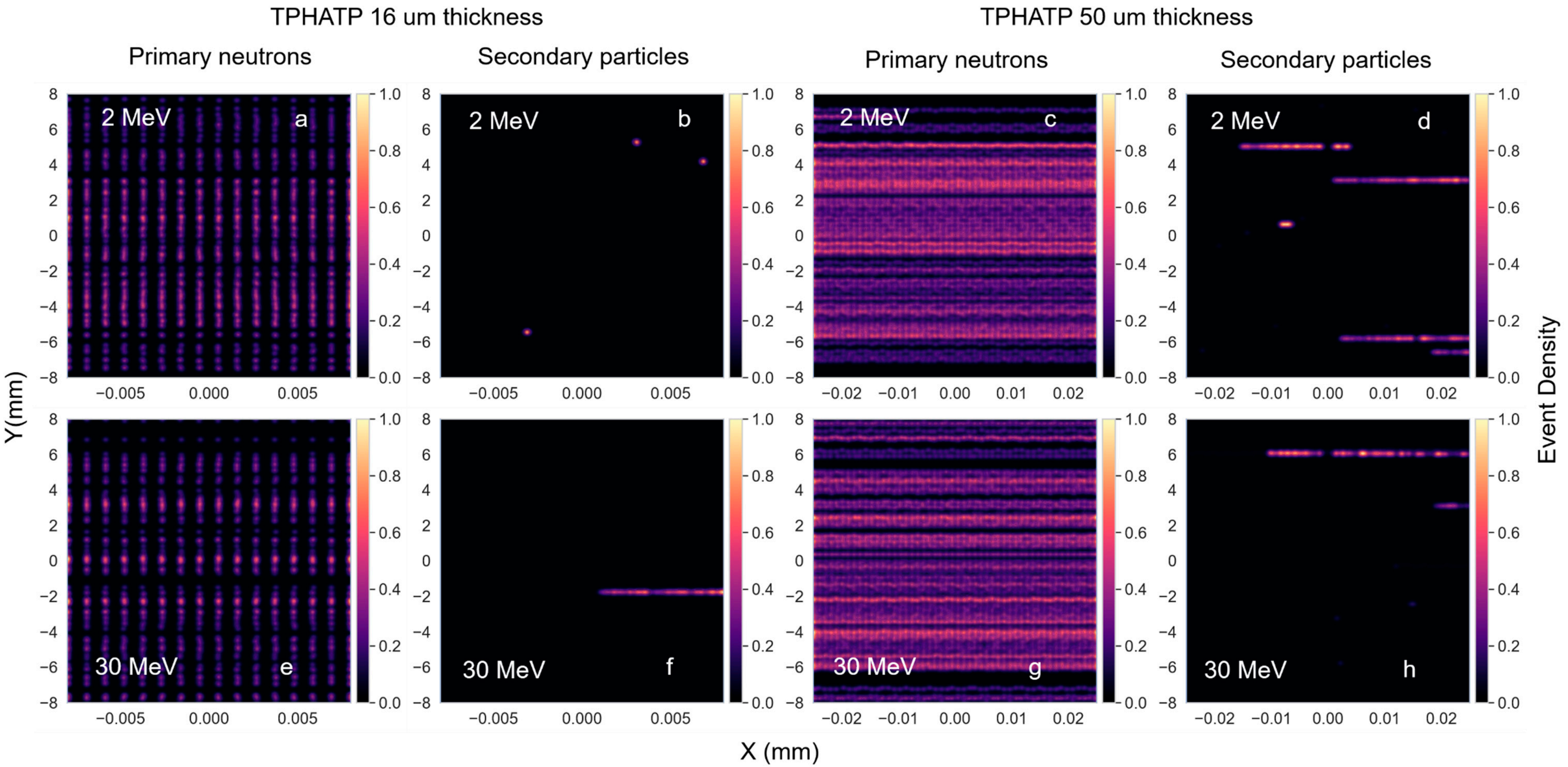

Figure S2**.** Event distribution maps in TPHATP layer of different thickness (16 um—a, b, e, f; 50 um—c, d, g, h) under 2 (a–d) and 30 (e–h) MeV neutron irradiation.

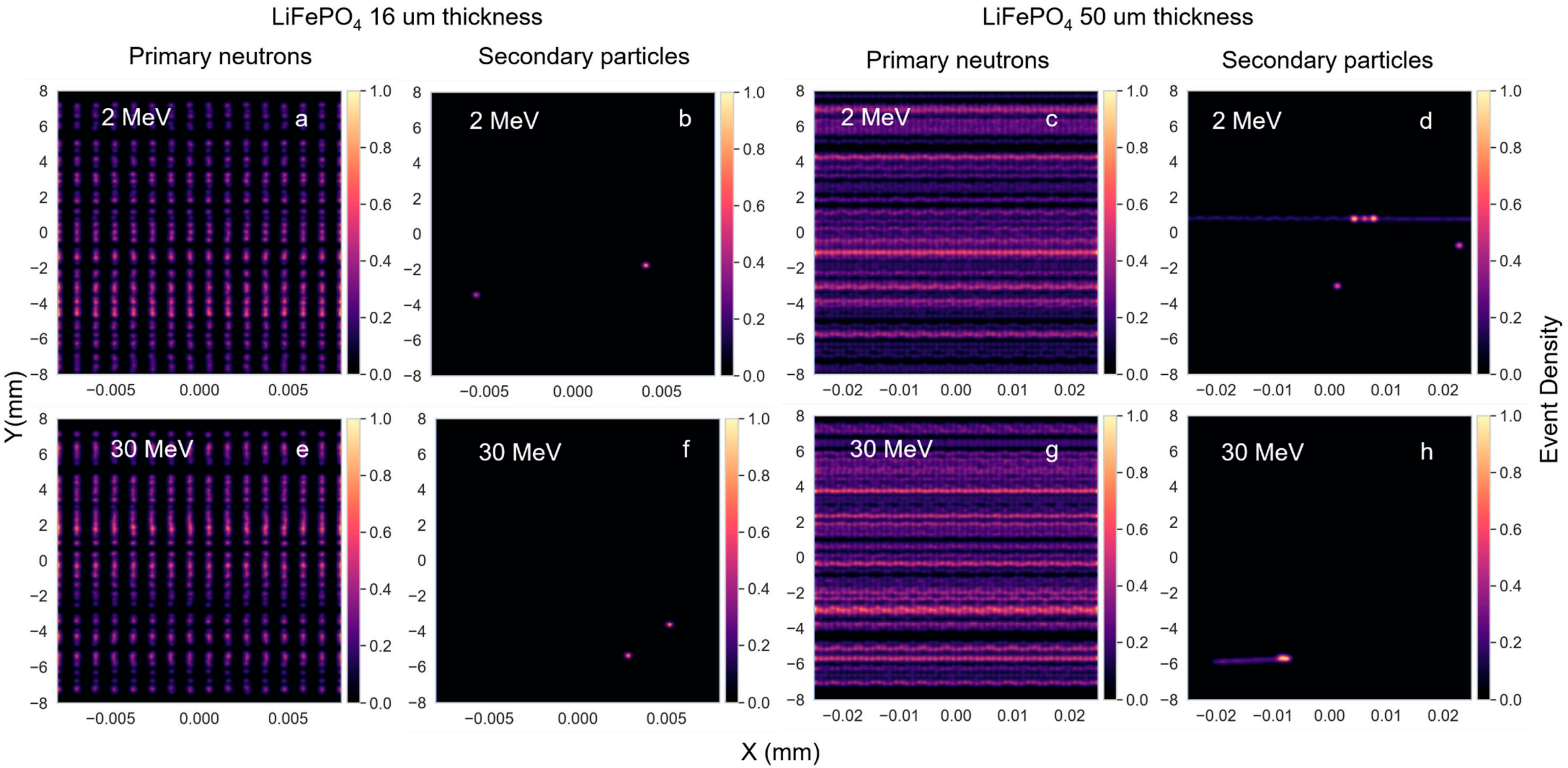

Figure S3**.** Event distribution maps in $LiFePO_4$ layer of different thickness (16 um—a, b, e, f; 50 um—c, d, g, h) under 2 (a–d) and 30 (e–h) MeV neutron irradiation.

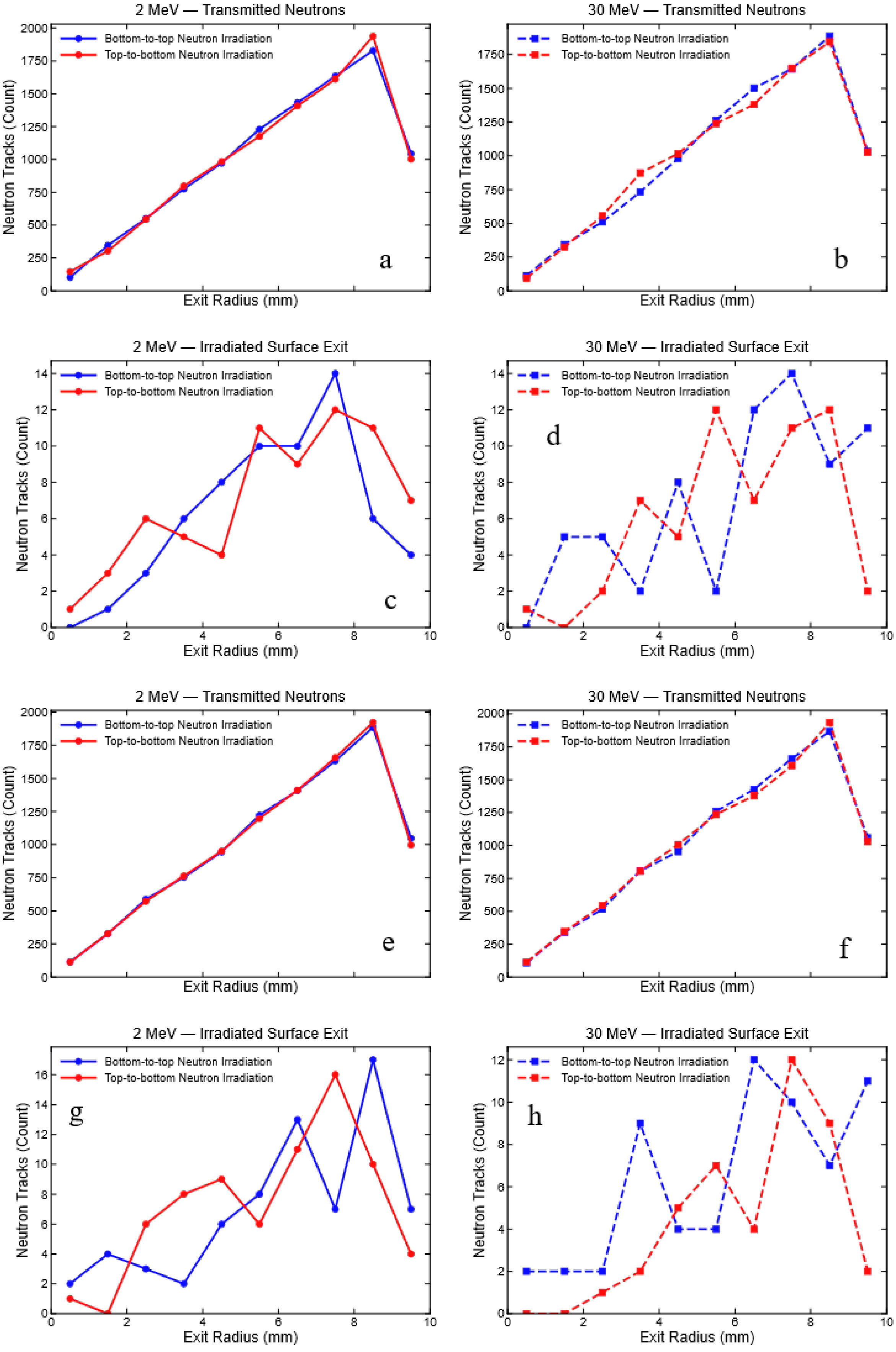

Figure S4. Radial distributions of neutron tracks exiting the TPHATP-based (a–d) and $LiFePO_4$-based (e–h) cells under monoenergetic irradiation at 2 and 30 MeV.

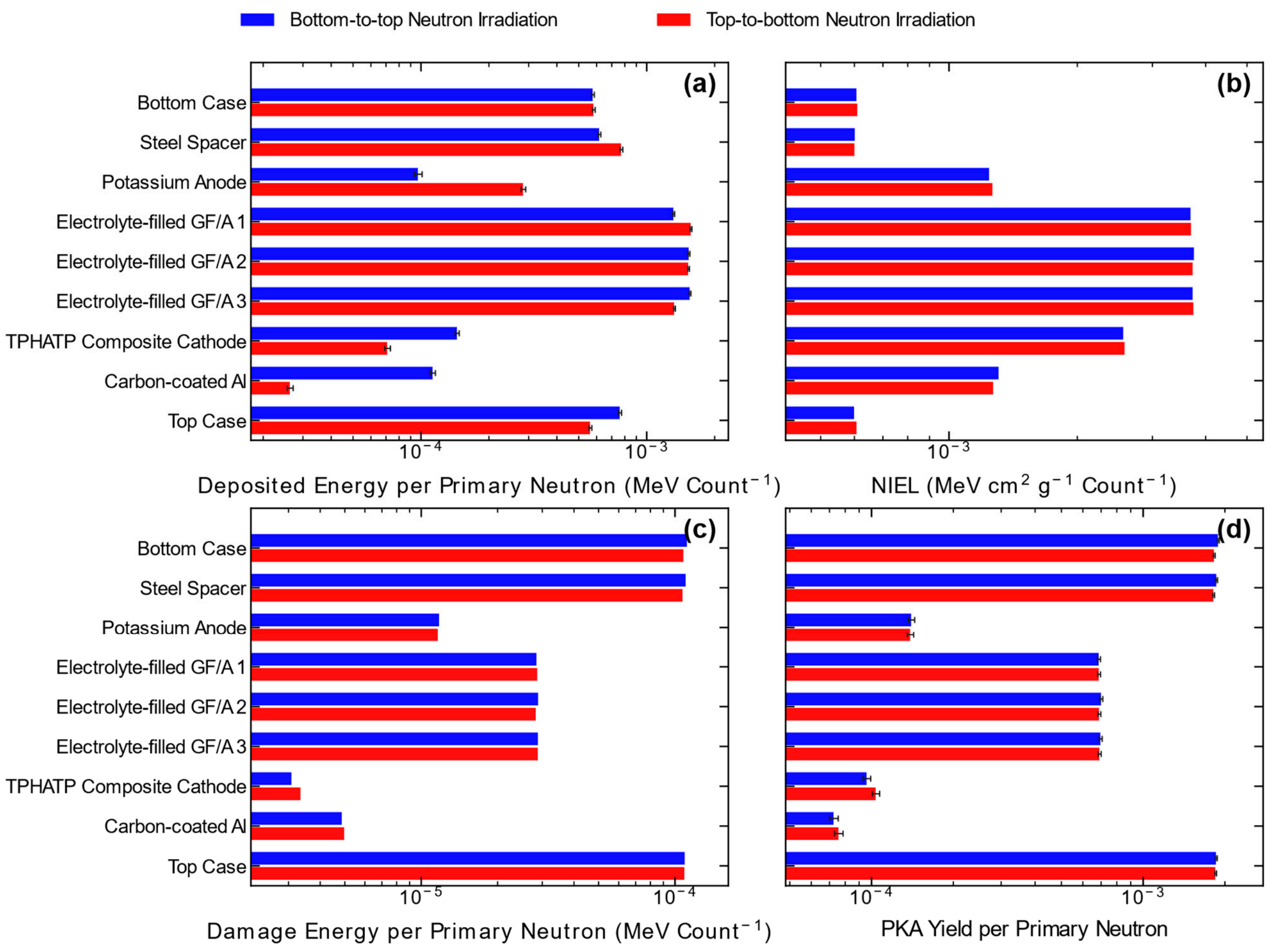

Figure S5. Layer-resolved response of the TPHATP-based CR2032 cell calculated using the JEFF-3.3 prompt fission neutron spectrum: (a) deposited energy per primary neutron, (b) non-ionizing energy loss (NIEL) per primary neutron, (c) damage energy per primary neutron, and (d) primary knock-on atom (PKA) yield per primary neutron. Error bars in panels (a) and (d) represent the statistical uncertainties of the Monte Carlo tallies.

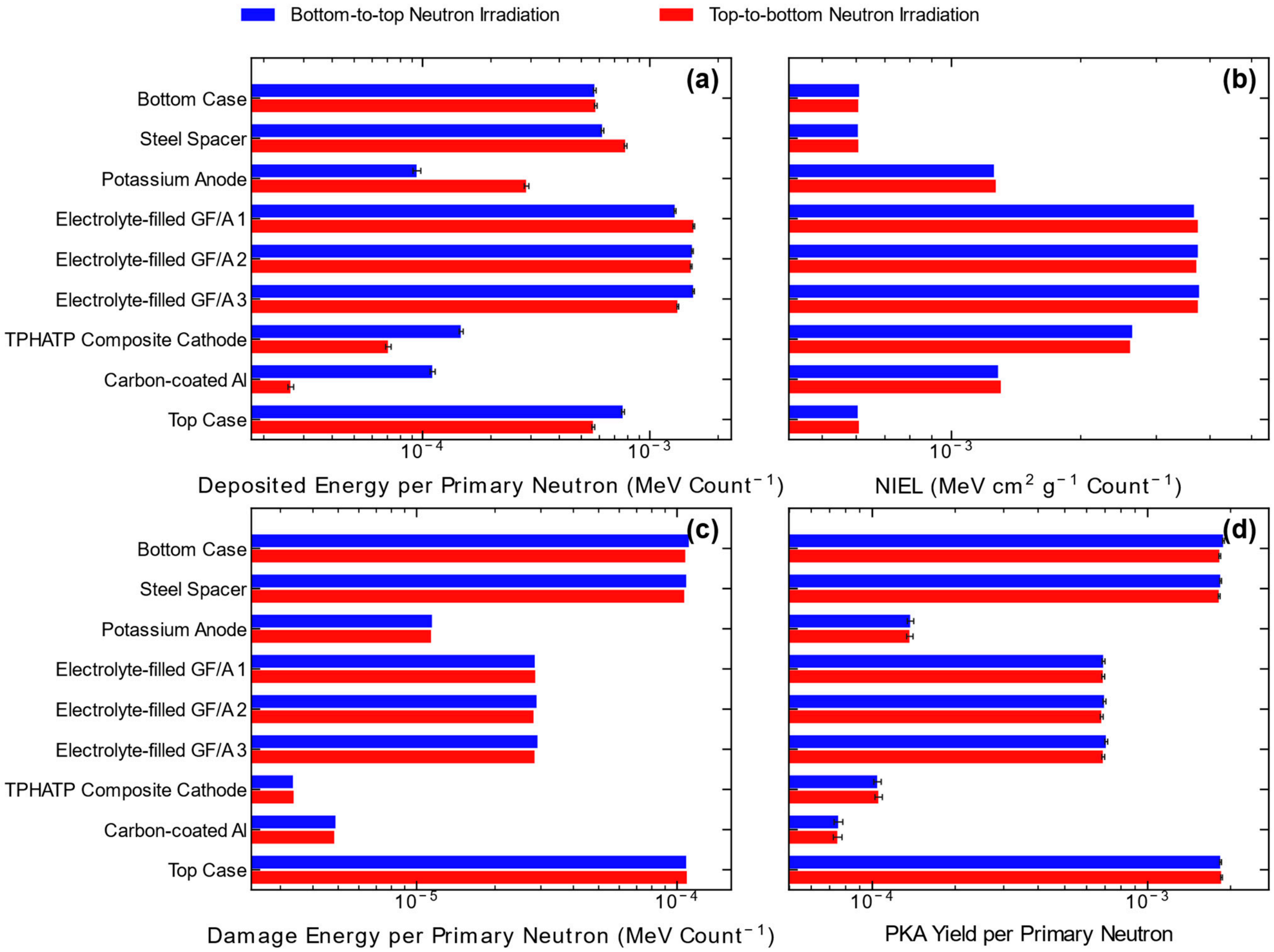

Figure S6. Layer-resolved response of the TPHATP-based CR2032 cell calculated using the JENDL-5 prompt fission neutron spectrum: (a) deposited energy per primary neutron, (b) non-ionizing energy loss (NIEL) per primary neutron, (c) damage energy per primary neutron, and (d) primary knock-on atom (PKA) yield per primary neutron. Error bars in panels (a) and (d) represent the statistical uncertainties of the Monte Carlo tallies.

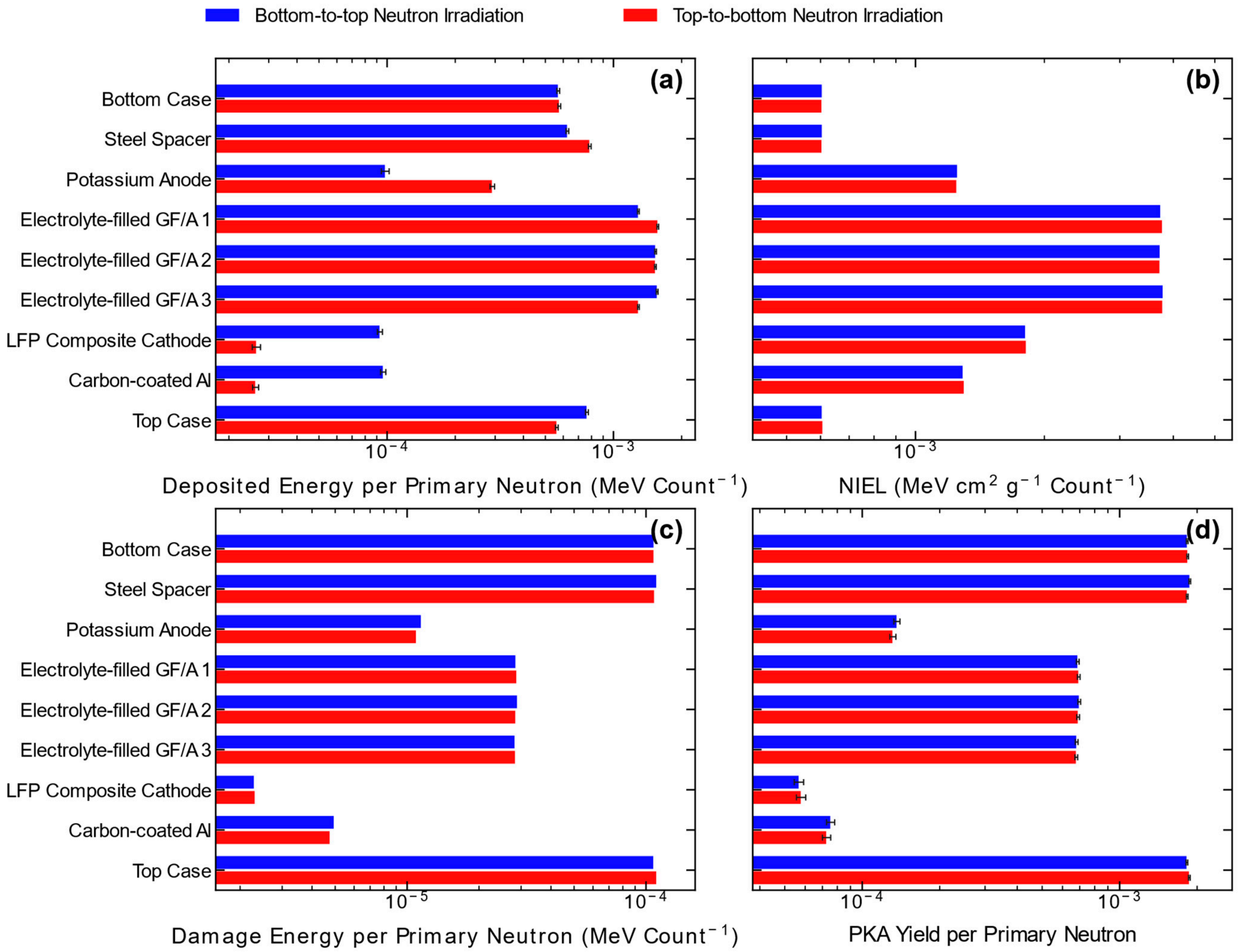

Figure S7. Layer-resolved response of the $LiFePO_4$-based (LFP) CR2032 cell calculated using the ENDF/B-VIII.0 prompt fission neutron spectrum: (a) deposited energy per primary neutron, (b) non-ionizing energy loss (NIEL) per primary neutron, (c) damage energy per primary neutron, and (d) primary knock-on atom (PKA) yield per primary neutron. Error bars in panels (a) and (d) represent the statistical uncertainties of the Monte Carlo tallies.

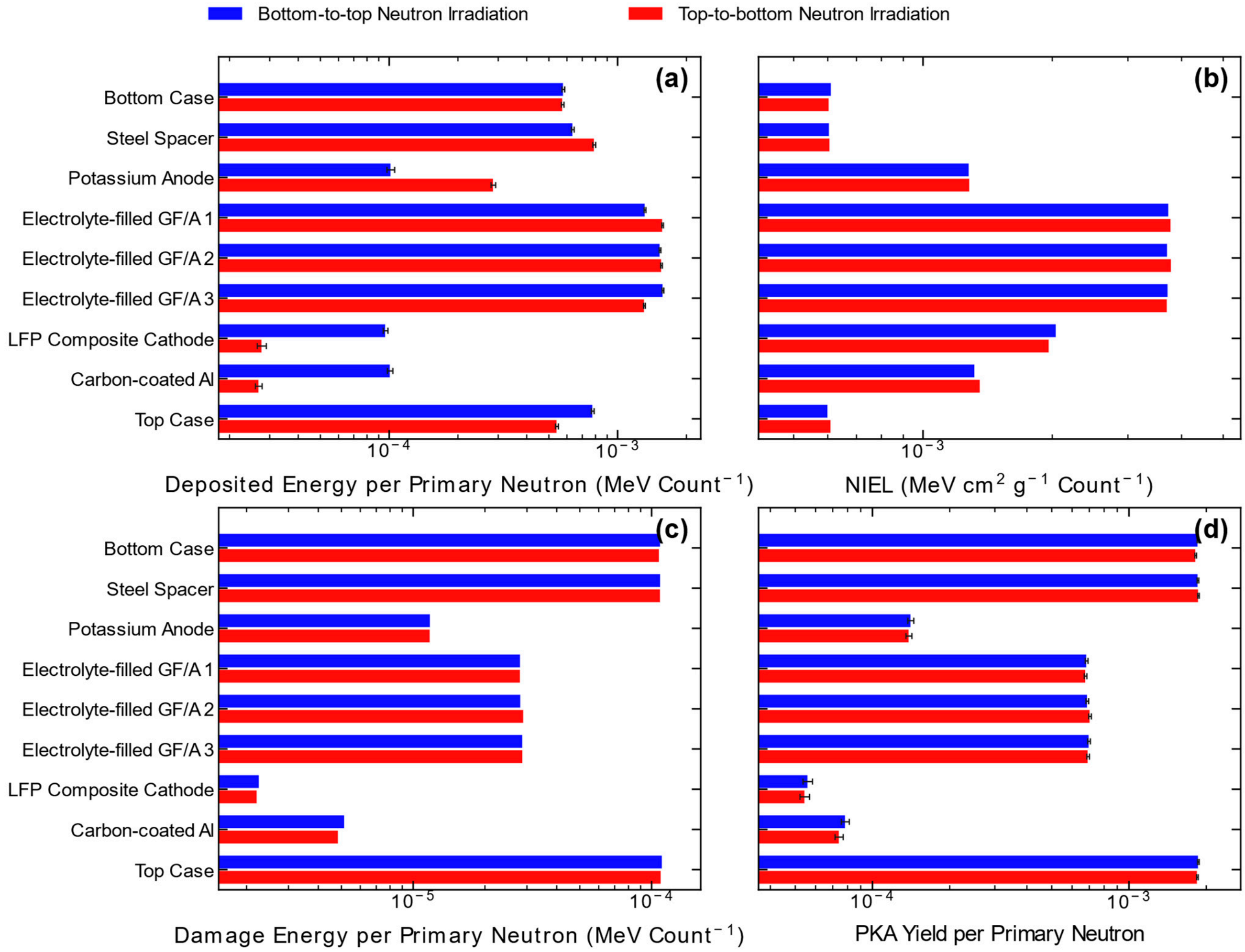

Figure S8. Layer-resolved response of the LiFePO4-based (LFP) CR2032 cell calculated using the JEFF-3.3 prompt fission neutron spectrum: (a) deposited energy per primary neutron, (b) non-ionizing energy loss (NIEL) per primary neutron, (c) damage energy per primary neutron, and (d) primary knock-on atom (PKA) yield per primary neutron. Error bars in panels (a) and (d) represent the statistical uncertainties of the Monte Carlo tallies.

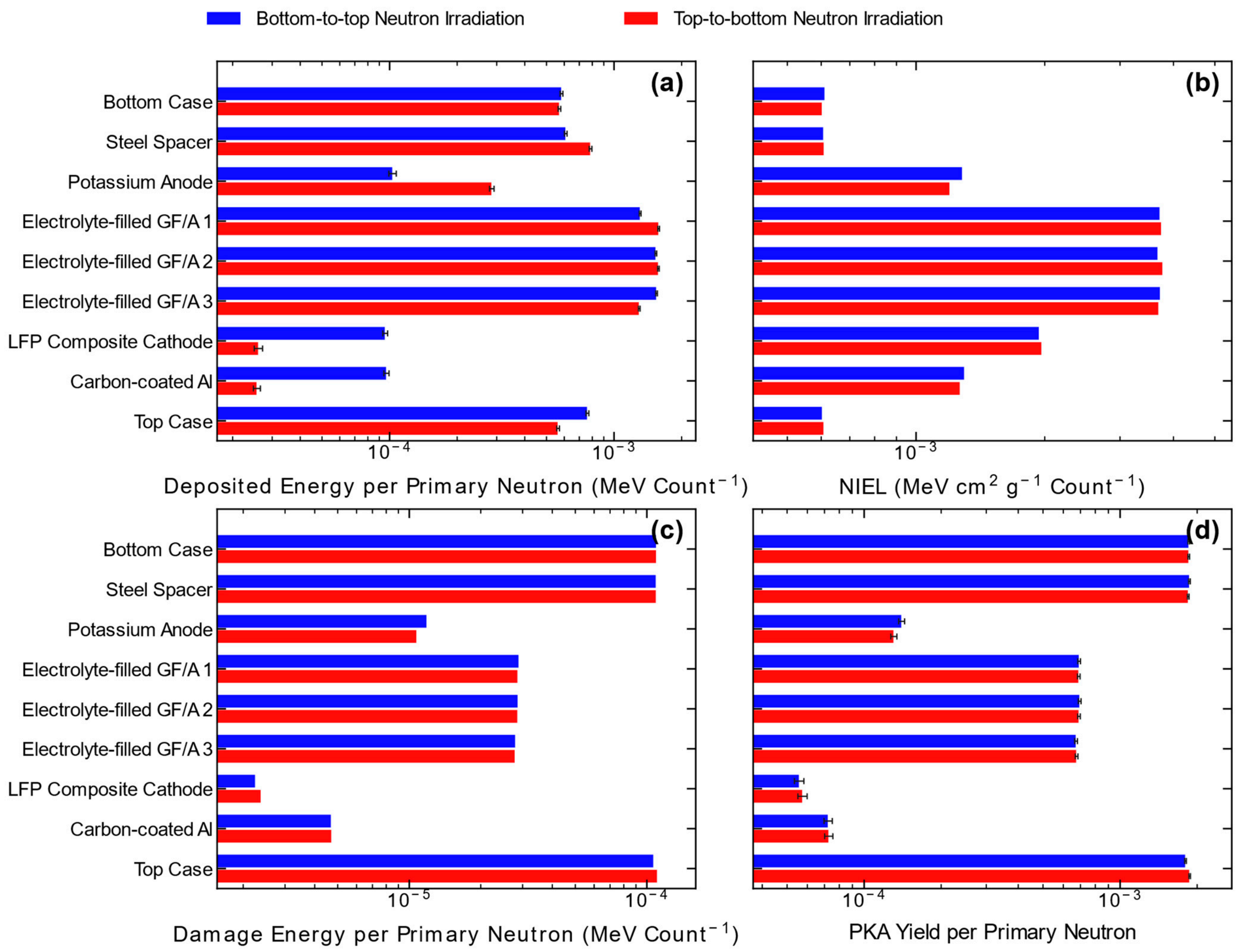

Figure S9. Layer-resolved response of the $LiFePO_4$-based (LFP) CR2032 cell calculated using the JENDL-5 prompt fission neutron spectrum: (a) deposited energy per primary neutron, (b) non-ionizing energy loss (NIEL) per primary neutron, (c) damage energy per primary neutron, and (d) primary knock-on atom (PKA) yield per primary neutron. Error bars in panels (a) and (d) represent the statistical uncertainties of the Monte Carlo tallies.

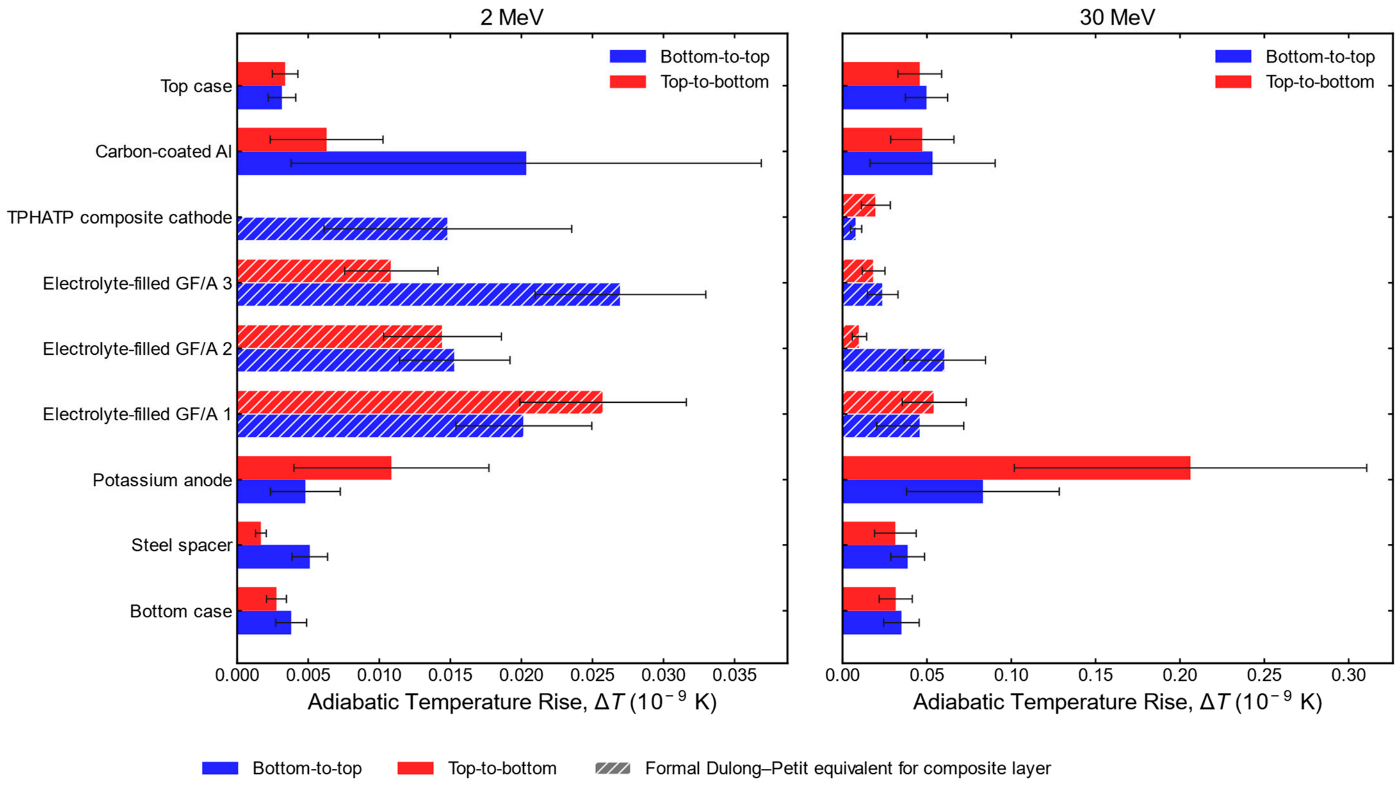

Figure S10. Normalized adiabatic temperature rise in TPHATP-based cell layers caused by generated secondary particles.

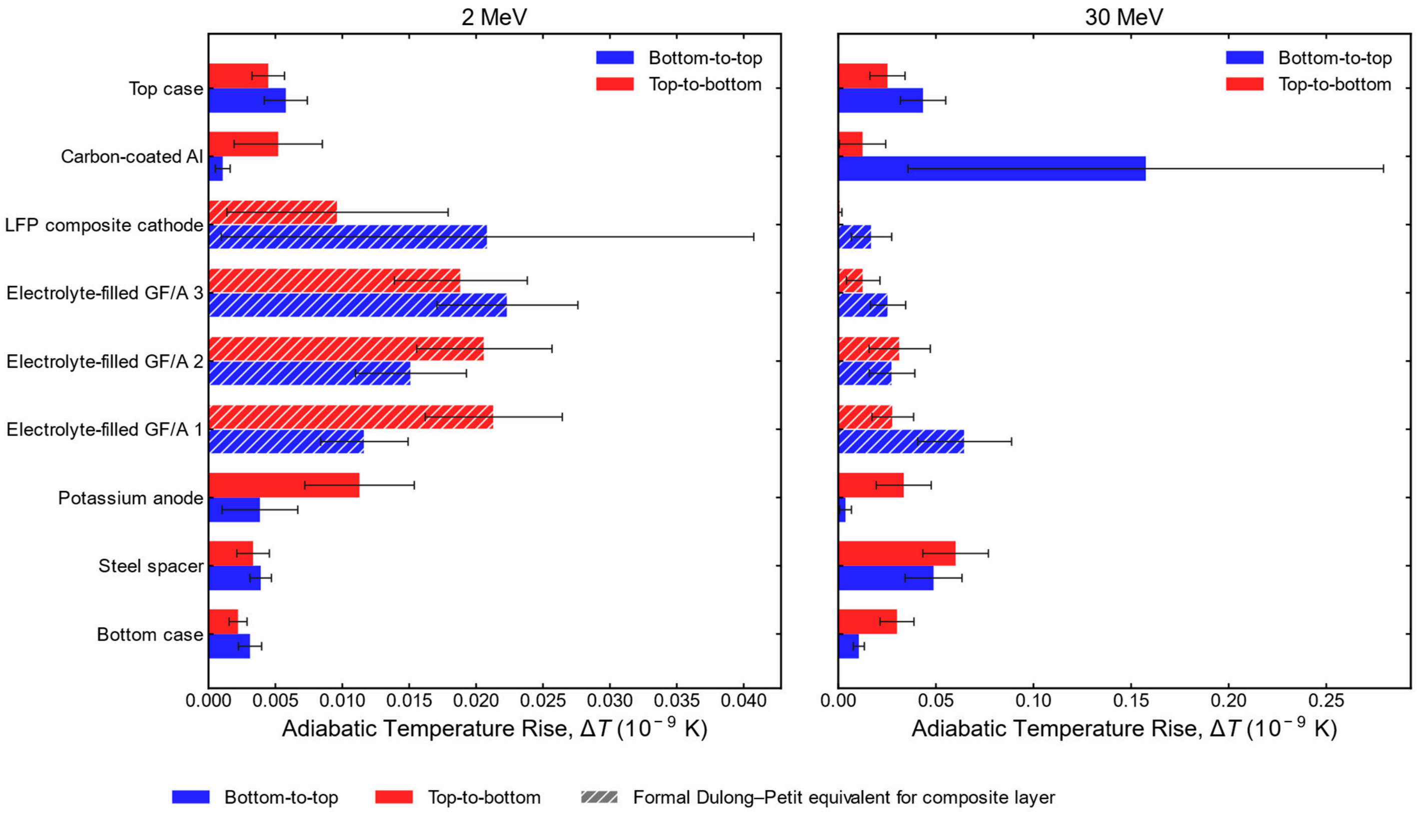

Figure S11. Normalized adiabatic temperature rise in LFP-based cell layers caused by generated secondary particles.

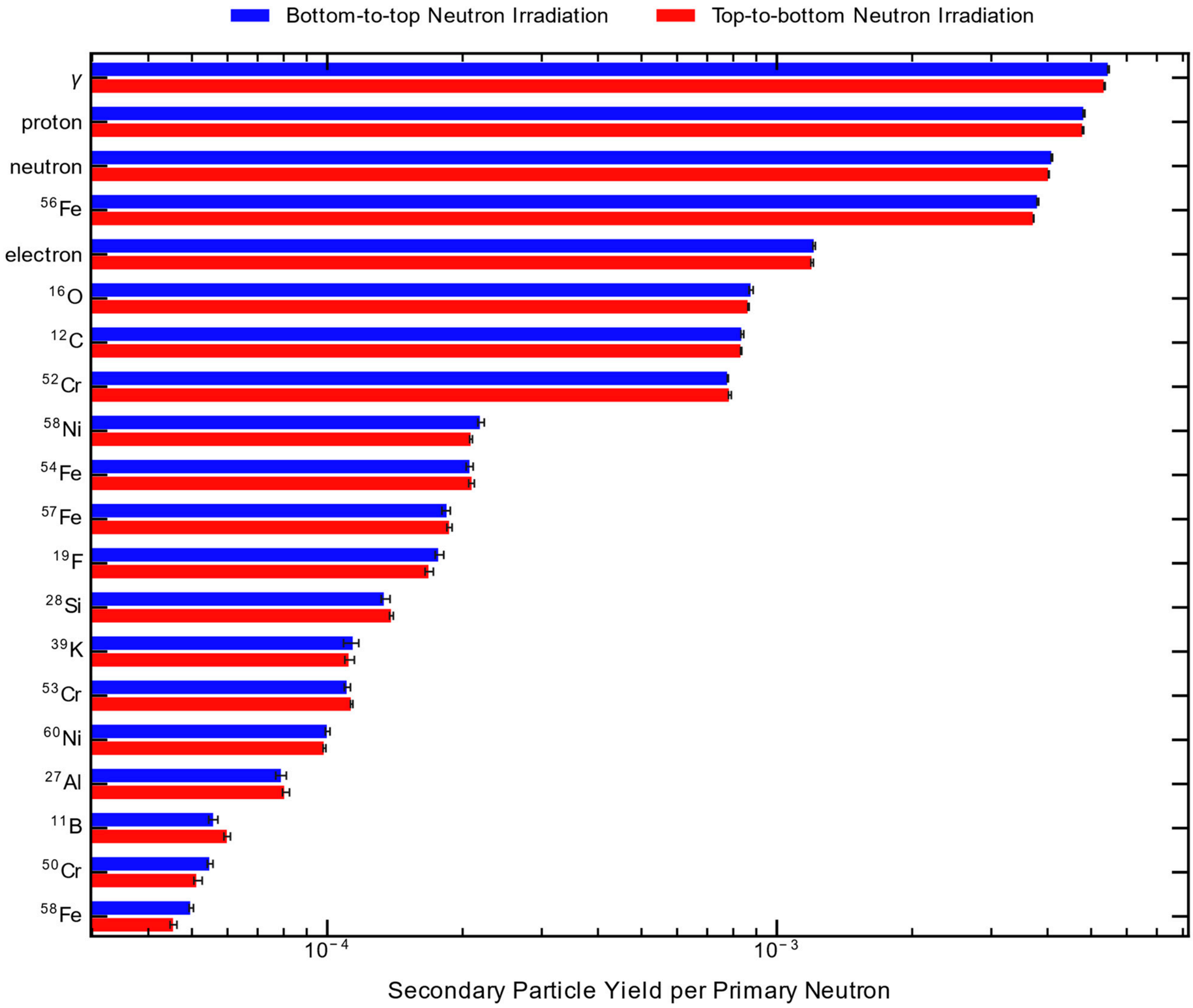

Figure S12. Secondary particle production in the TPHATP-based CR2032 cell under bottom-to-top and top-to-bottom irradiation.

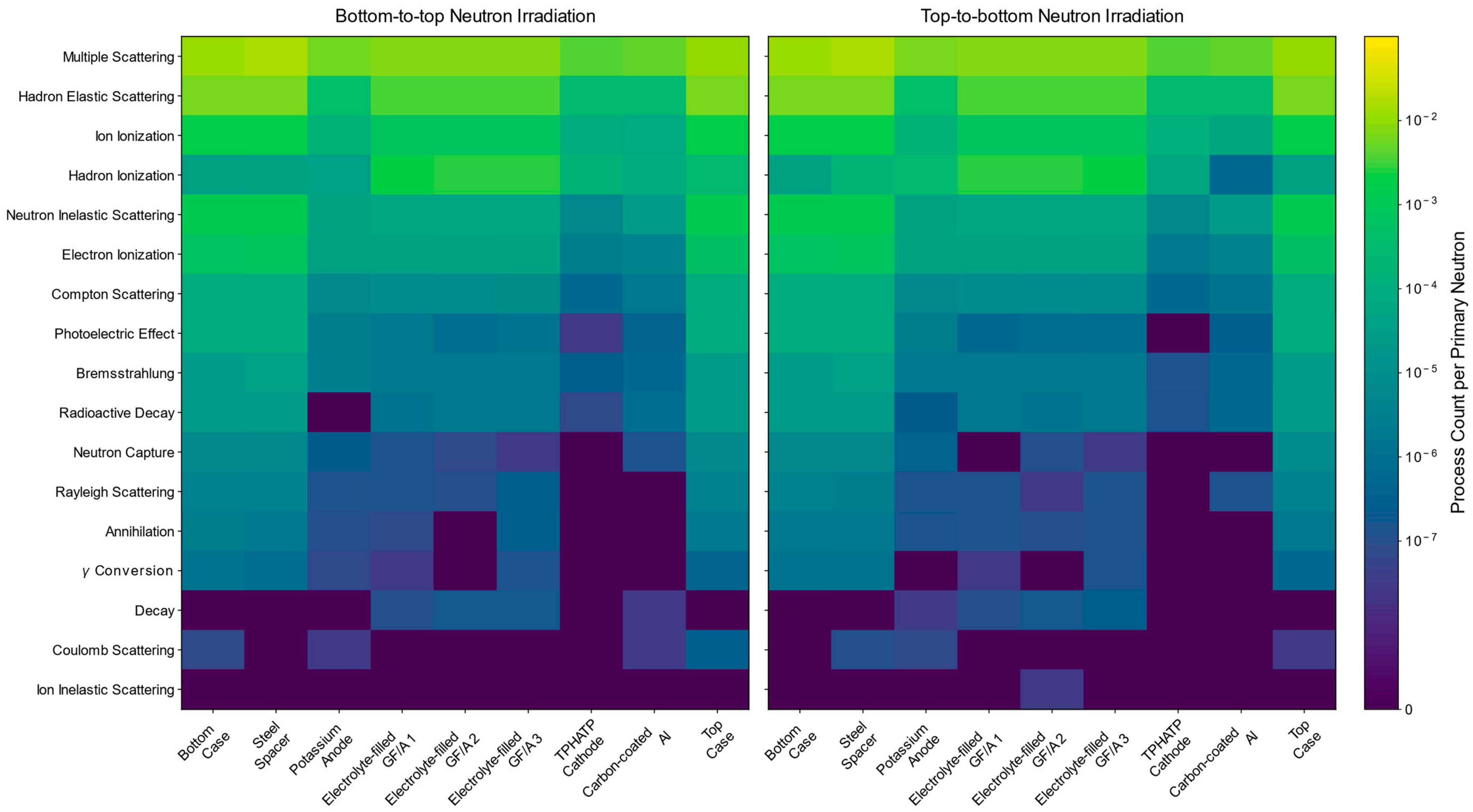

Figure S13. Interaction process distribution in the TPHATP-based CR2032 cell under bottom-to-top and top-to-bottom irradiation.

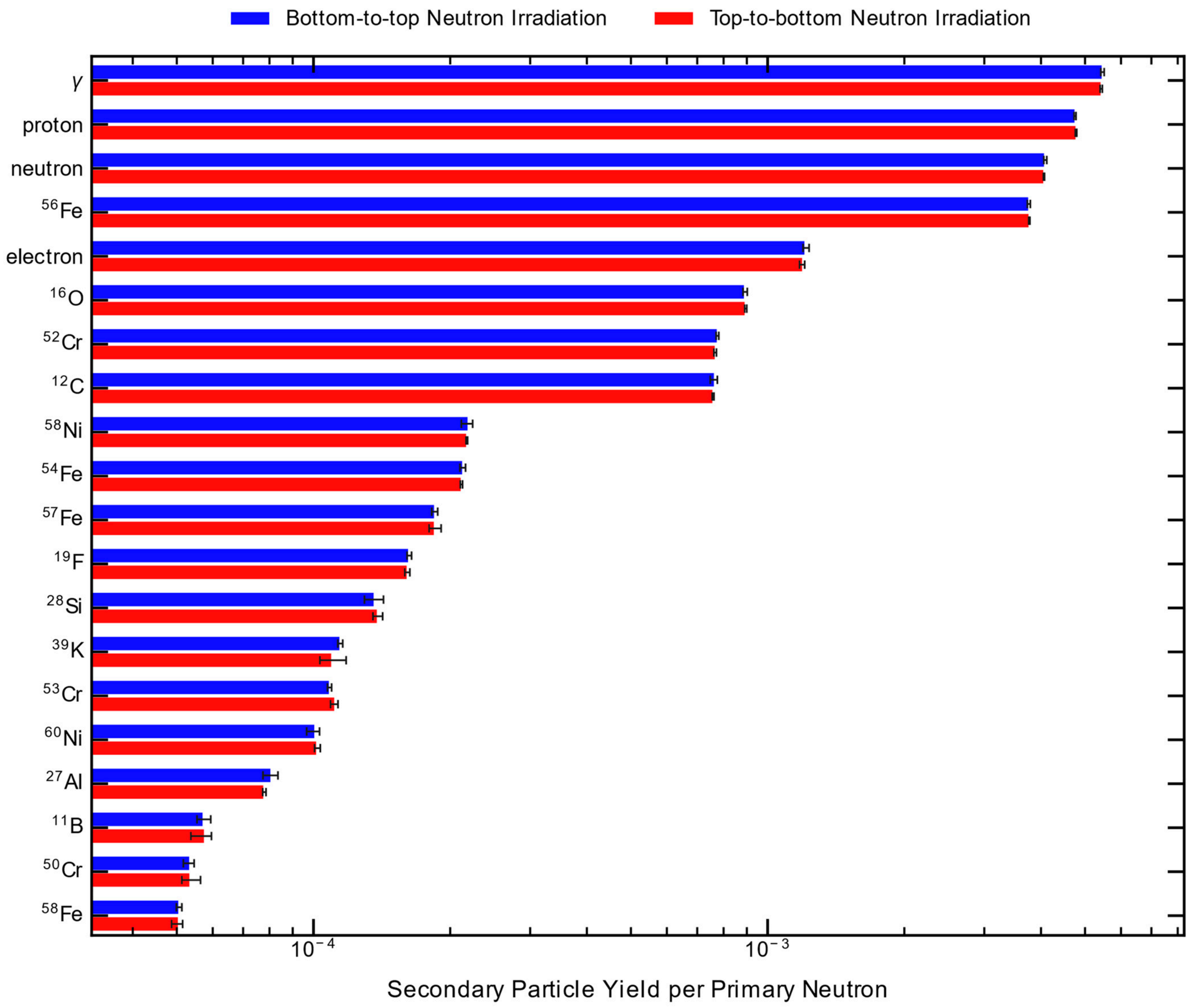

Figure S14. Secondary particle production in the LFP-based CR2032 cell under bottom-to-top and top-to-bottom irradiation.

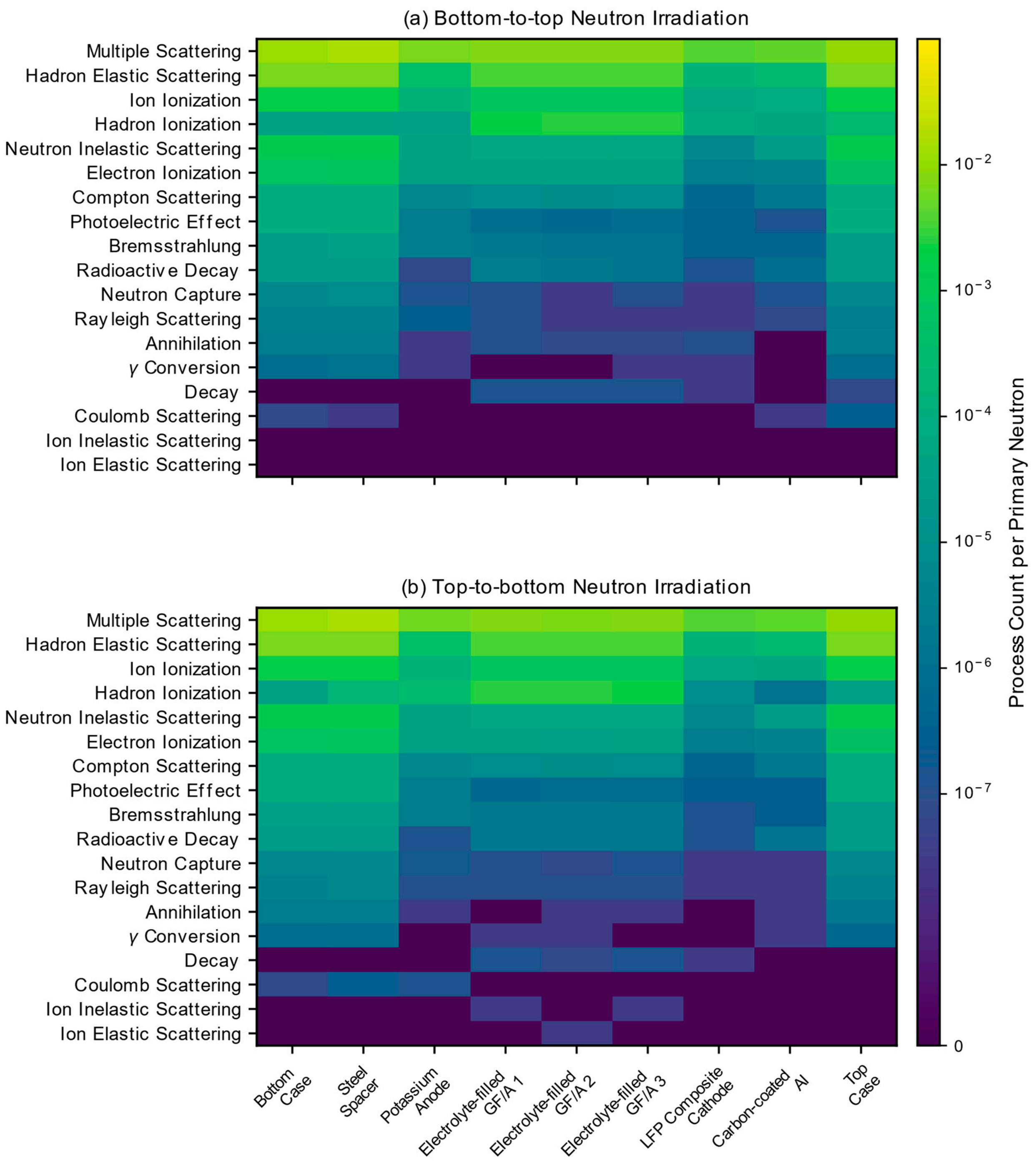

Figure S15. Interaction process distribution in the LFP-based CR2032 cell under bottom-to-top and top-to-bottom irradiation.

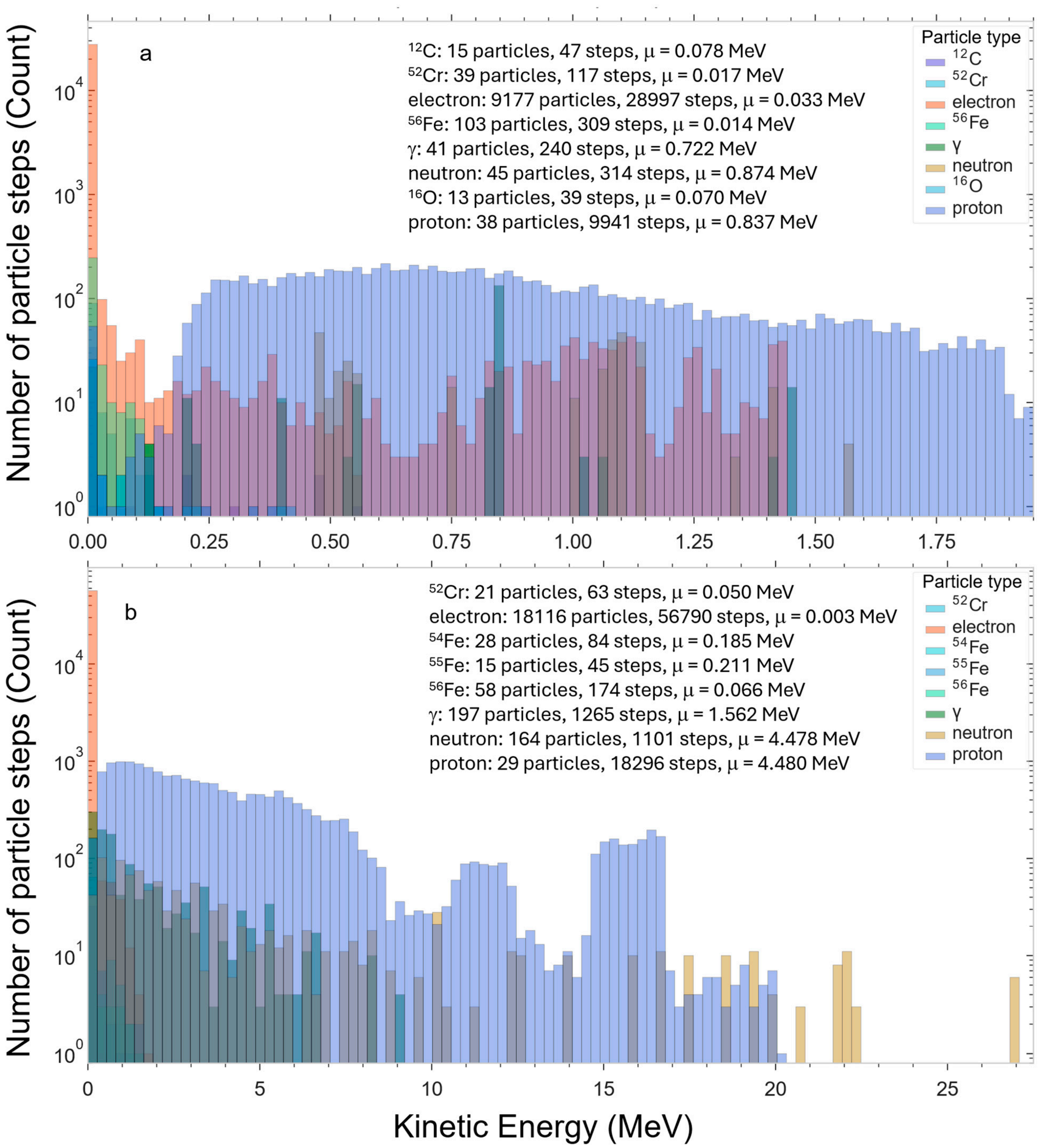

Figure S16. Energy distributions of secondary particles produced in the LFP-based CR2032 cell under monoenergetic neutron irradiation at (a) 2 MeV and (b) 30 MeV. The annotations report the number of recorded particles, the number of transport steps, and the mean kinetic energy μ for each selected species.

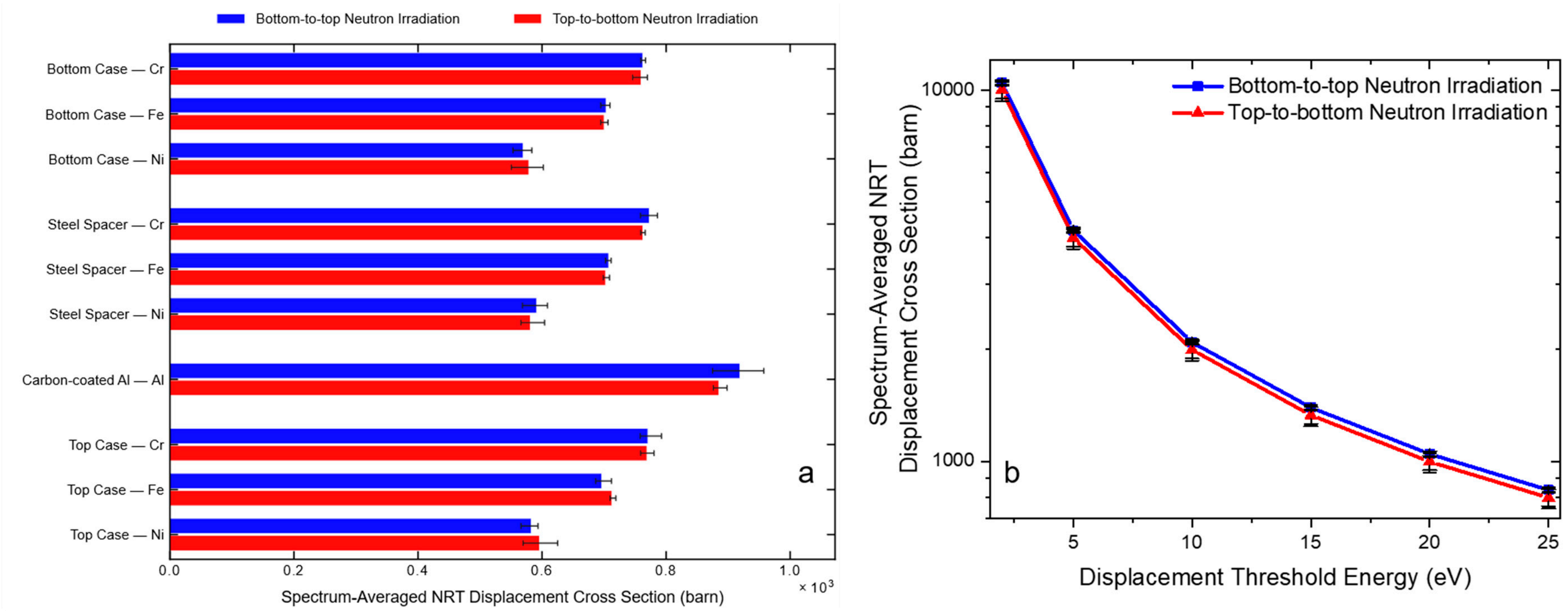

Figure S17. Spectrum-averaged NRT displacement response of selected cell materials in LFP-based battery: (a) displacement cross-sections for Cr, Fe, Ni, and Al in the metallic cell components, (b) dependence of the spectrum-averaged displacement cross-section of metallic potassium on the assumed threshold displacement energy under bottom-to-top and top-to-bottom irradiation.
